# Towards generalizable seizure monitoring: EpiVLM for cross-environment detection and classification

**DOI:** 10.1038/s41746-026-02810-3

**Published:** 2026-05-26

**Authors:** Mengqiao He, Leihao Sha, Guoling Tang, Jinguo Pang, Ling Jin, Yutong Fu, Sikai Huang, Wentao Wang, Shixian Wen, Yi Yao, Pengfei Wei, Lei Chen

**Affiliations:** 1 Department of Neurology, West China Hospital, Sichuan University, Chengdu, Sichuan China; 2 Sichuan Provincial Engineering Research Center of Brain-machine Interactive Neuromodulation, Chengdu, Sichuan China; 3 Laboratory of Neuro-diseases and Multimorbidity, West China Hospital, Sichuan University, Chengdu, Sichuan China; 4 Sichuan Provincial Model Worker and Craftsman Innovation Studio, Chengdu, Sichuan China; 5 School of Biological Science and Medical Engineering, State Key Laboratory of Digital Medicine, Southeast University, Nanjing, China; 6 Shenzhen Institutes of Advanced Technology, Chinese Academy of Sciences, Shenzhen, China; 7 Southern University of Science and Technology, Shenzhen, China; 8 China Telecom Corporation Limited Sichuan Branch, Chengdu, China; 9 Guangdong Provincial Key Laboratory of Brain Connectome and Behavior, the Brain Cognition and Brain Disease Institute, Shenzhen Institutes of Advanced Technology, Chinese Academy of Sciences, Shenzhen, China; 10 CAS Key Laboratory of Brain Connectome and Manipulation, Shenzhen - Hong Kong Institute of Brain Science, Shenzhen Institutes of Advanced Technology, Chinese Academy of Sciences, Shenzhen, China; 11 Brain Everest LLC, Shenzhen, China; 12 Department of Functional Neurosurgery, Xiamen Humanity Hospital, Fujian Medical University, Fuzhou, China

**Keywords:** Computational biology and bioinformatics, Health care, Medical research, Neurology, Neuroscience

## Abstract

The translation of automated seizure detection from controlled clinical units to real-world settings is hindered by heterogeneous recording conditions and limited expert monitoring. We introduce EpiVLM, a multimodal vision–language system that combines clinically structured prompts with video reasoning for cross-environment seizure monitoring. Evaluated on a robust and diverse dataset of 232 video recordings from 127 patients, totaling 11,666 expert-annotated segments from two tertiary centers, unconstrained home recordings, and an independent public dataset, EpiVLM recognized five major semiologies with accuracy 0.795–0.947 and sensitivity 0.842–0.957. With prompts and decision thresholds fixed a priori, performance remained consistent across diverse real-world acquisition conditions without site-specific recalibration. In external validation sets, EpiVLM sustained strong recognition while maintaining low video-level false detections (0.47–2.45%) and timely detection (mean onset-to-detection delay <6 s). Compared with standard video deep-learning baselines, EpiVLM achieved superior overall performance. These results support scalable seizure recognition from routine video and motivate prospective evaluation for remote outcome monitoring.

## Introduction

Epilepsy is one of the most prevalent and debilitating neurological disorders worldwide, affecting more than 50 million people across all age groups, with a particularly heavy burden in low- and middle-income countries (LMICs)^1^. Beyond recurrent, unprovoked seizures, patients face profound cognitive and psychosocial comorbidities, as well as heightened risks of injury and sudden unexpected death in epilepsy (SUDEP)^2–4^. Despite substantial advances in neuroimaging, pharmacological therapies, and surgical interventions, seizure detection and timely management remain inconsistent^5–8^. Strikingly, more than half of seizures globally are under-recognized or misclassified, leading to preventable mortality and misguided therapeutic decisions^9,10^. These challenges are particularly acute in LMICs, where shortages of specialized neurologists and limited availability of long-term video-EEG monitoring persist^11,12^.

Accurate and scalable seizure detection in real-world settings represents a persistent and critical unmet need in epilepsy management^13,14^. Conventional approaches, such as patient or caregiver self-reports^15^ and inpatient video-electroencephalography^16,17^ (video-EEG), have substantial limitations^18^. Self-reports are often inaccurate or incomplete due to recall bias, limited health literacy, or unobserved events^19^. While video-EEG remains the diagnostic gold standard, it requires prolonged inpatient monitoring, costly equipment, and continuous expert interpretation, making it impractical for long-term or home-based surveillance^20^. Outpatient or smartphone video recordings have emerged as adjunct tools for diagnosis^21^; however, they still depend on manual review by specialists, creating bottlenecks in scalability and delaying timely intervention^9,21^.

Recent advances in deep learning have catalyzed progress in video-based seizure detection, but current approaches remain limited in clinical applicability. Many models are trained and validated primarily on generalized tonic-clonic seizures, assuming standardized hospital conditions that do not reflect the variability of real-world environments^22–24^. Others depend on motion-capture systems or specialized wearable sensors, which increase complexity and reduce accessibility for patients and caregivers outside tertiary care centers^25,26^. Moreover, the limited size and homogeneity of most existing datasets hinder robust generalization across diverse patient populations, seizure semiologies, and environmental contexts^27^. These challenges have prevented artificial intelligence (AI)-assisted seizure monitoring from fully transitioning into reliable, scalable, and practical clinical solutions. Beyond these limitations, pose-based pipelines^28–30^ (2D/3D keypoints) are fragile in routine wards: blankets occlude limbs, multi-person scenes cause identity switching, and variable viewpoints degrade keypoint estimation. Many require patient-specific calibration or repeated annotation, further limiting scalability for continuous, real-world monitoring.

To bridge these gaps, we developed EpiVLM (Epilepsy Vision–Language Model System), a multimodal vision–language model (VLM)-based platform designed for real-time, automated detection and classification of epileptic seizures across a broad range of environments—from hospital monitoring units to patients’ homes and workplaces. Leveraging state-of-the-art multimodal AI architectures, the system integrates patient segmentation with SAM2 (Segment Anything Model 2)^31^, domain-specific prompt engineering, and supervised fine-tuning of the Qwen2.5-VL-32B backbone^32^. This integrated pipeline enables accurate identification and classification of diverse seizure types while maintaining robust performance under challenging conditions, such as partial occlusions, variable lighting, and multi-person interactions.

**Fig. 1:**
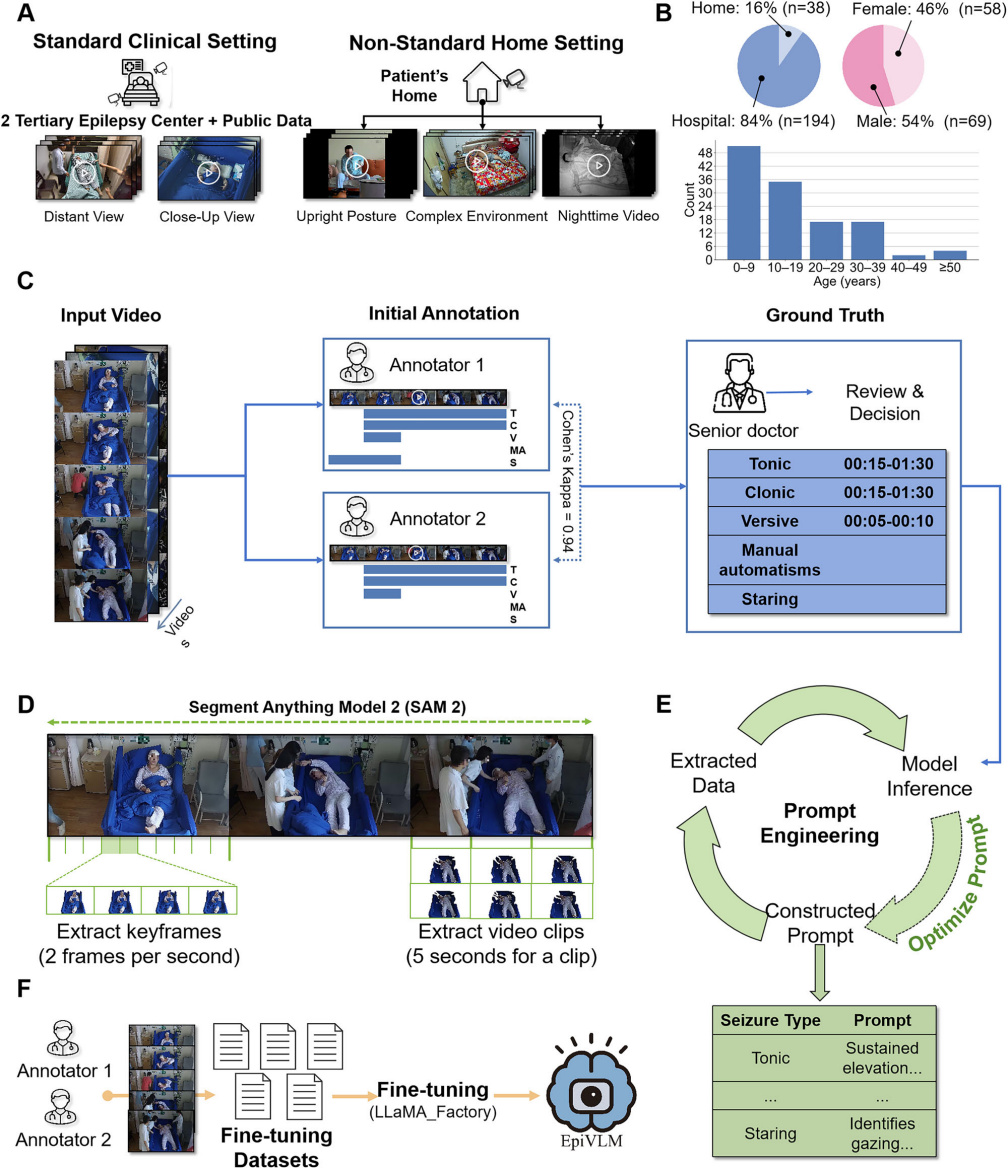
Workflow of the EpiVLM system for automated seizure detection and classification. **A** Data were collected from two tertiary epilepsy centers, a public dataset, and home recordings, capturing diverse perspectives. **B** A total of 232 videos from 127 patients (54% male, 46% female) were categorized by age in 10-year intervals, with the largest group being 0–9 years and the smallest 40–49 years. Patients over 50 were grouped together. **C** Video data were annotated every 5 s by two trained neurologists across five seizure types, with final validation by a senior doctor, forming the Ground Truth dataset. **D** The SAM2 model segmented epilepsy patients from the videos, extracting key frames and clips. **E** Key frames/clips were processed with specific prompts for each seizure type, optimized through prompt engineering, and inferred by a large language model. **F** Fine-tuning datasets for each seizure type were created by annotators, and the LLaMA_Factory framework was used to fine-tune the model, resulting in EpiVLM.

In this study, we developed and evaluated EpiVLM using a large, multicenter cohort spanning from toddlers to adults. While our dataset predominantly comprises pediatric and younger adult patients, it captures substantial real-world heterogeneity in seizure semiologies and monitoring environments essential for robust validation. The overall workflow of the proposed system is illustrated in Fig. 1. In our evaluation, the platform demonstrated consistent accuracy, low-latency detection, and stable inference. These results suggest that EpiVLM has the potential to serve as a scalable tool for continuous remote monitoring, early caregiver alerts, and integration with electronic health records (EHRs). By translating multimodal artificial intelligence into a practical framework, this work provides a foundational step toward data-driven precision epilepsy care, while also establishing the groundwork for future validation in broader, age-diverse populations.

## Results

### Dataset characteristics and real-world variability

**Fig. 2:**
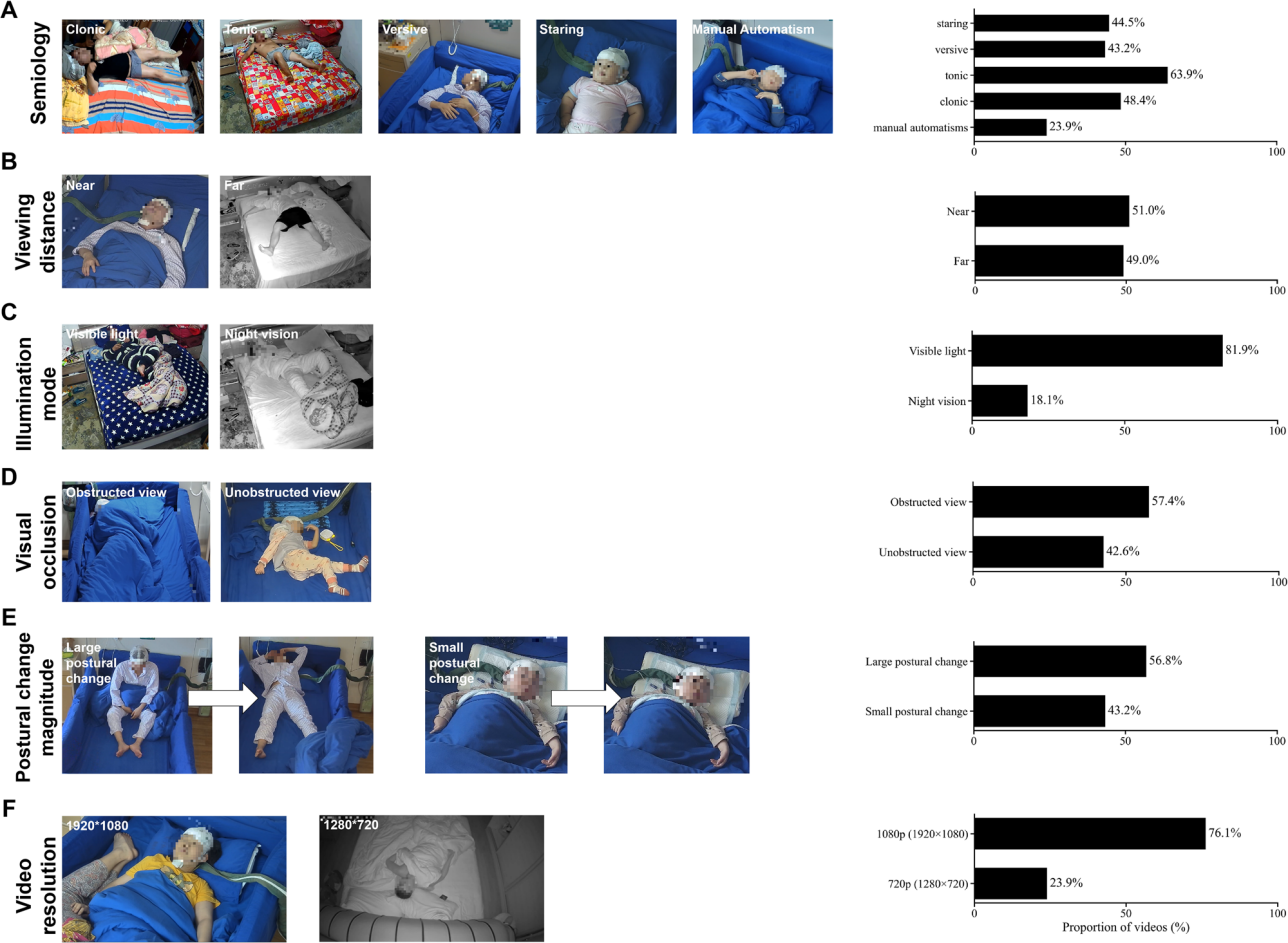
Real-world variability of recording conditions in the internal dataset. **A** Seizure semiologies and recording-condition categories. (**B**) viewing distance, (**C**) illumination mode, (**D**) visual occlusion, (**E**) postural-change magnitude, and (**F**) video resolution. Percentages indicate the proportion of videos in each category.

The internal dataset comprised 156 videos (7608 5-s clips) from 71 unique patients, covering five seizure semiologies—clonic, tonic, versive, manual automatisms, and staring—and displayed marked heterogeneity in real-world recording conditions. Viewing distance was nearly balanced (Near 51.0% vs Far 49.0%). By contrast, other factors were unevenly distributed: illumination (visible light 81.9% vs night vision 18.1%), visual occlusion (obstructed 57.4% vs unobstructed 42.6%), postural-change magnitude (large 56.8% vs small 43.2%), and resolution (1080p 76.1% vs 720p 23.9%) (Fig. 2A–F).

### Expert annotation reliability and human benchmark performance

Expert annotation was highly consistent across all five seizure semiologies, with Cohen’s κ ranging from 0.950 to 0.988 (Table 1). Agreement was highest for staring (κ = 0.988, 95% CI [0.984, 0.991]) and remained comparably strong for clonic (0.981, [0.976, 0.987]) and tonic (0.975, [0.969, 0.981]), while versive (0.950, [0.938, 0.959]) and manual automatisms (0.951, [0.942, 0.960]) showed slightly lower agreement.

**Table 1:** Inter-rater reliability and human vs EpiVLM performance by seizure semiology

Symptom	Inter-Rater Kappa (95% CI)	Human Average F1	EpiVLM Model F1	Human Average FPR	EpiVLM Model FPR
Clonic	0.981 (0.976, 0.987)	0.986	0.856	0.0038	0.0928
Manual Automatisms	0.951 (0.942, 0.960)	0.971	0.884	0.0028	0.2906
Staring	0.988 (0.984, 0.991)	0.991	0.960	0.0023	0.0907
Tonic	0.975 (0.969, 0.981)	0.983	0.850	0.0039	0.065
Versive	0.950 (0.938, 0.959)	0.964	0.849	0.0038	0.0538

**Fig. 3:**
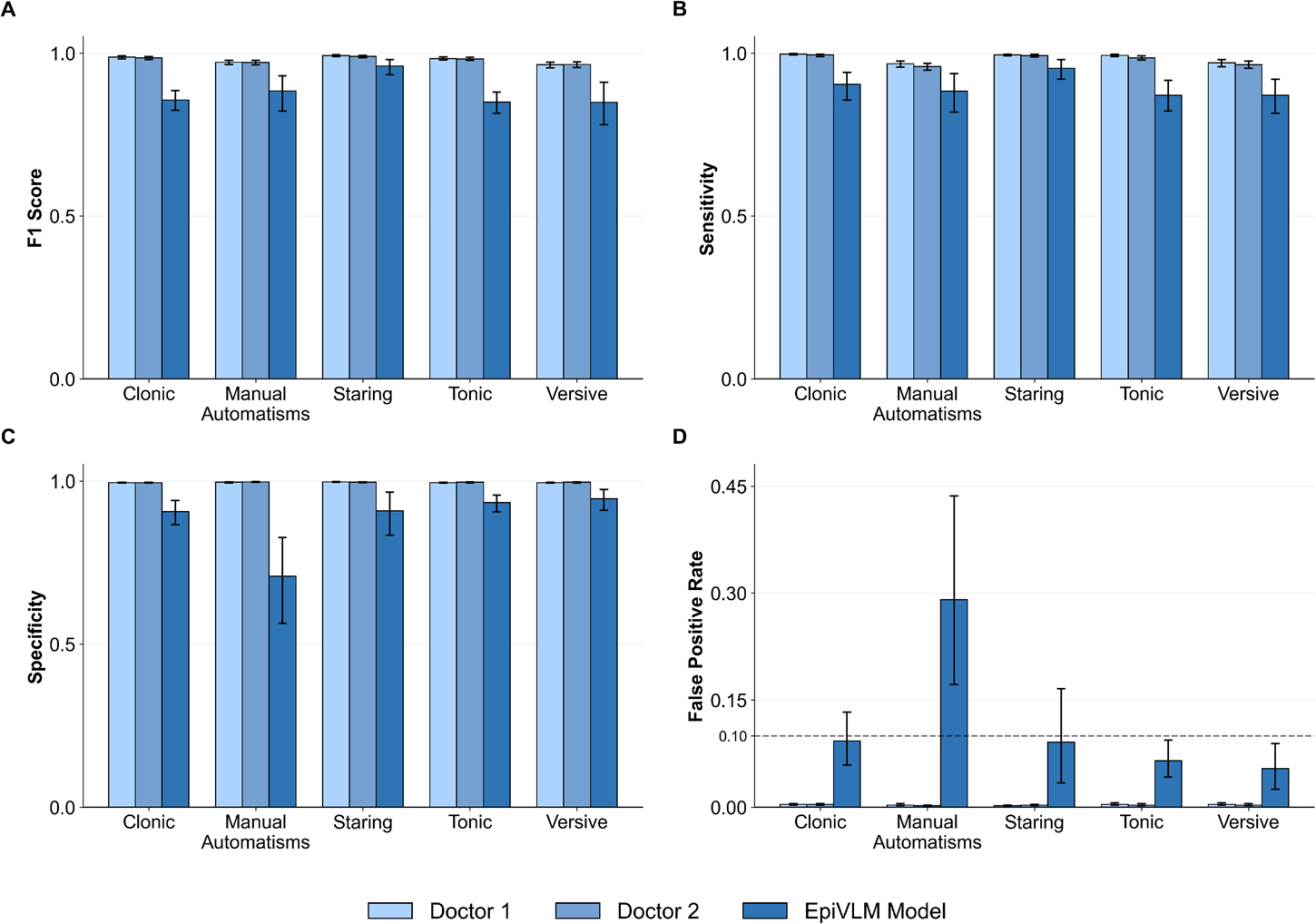
Comparison of human rater performance and EpiVLM across seizure semiology. Performance is stratified by seizure semiology (clonic, manual automatisms, staring, tonic, versive) and reported for two independent clinicians (Doctor 1 and Doctor 2) versus the EpiVLM model. **A** F1 score, (**B**) Sensitivity, (**C**) Specificity, (**D**) False positive rate (FPR).

Against the adjudicated reference labels, the two clinicians achieved near-ceiling performance with tightly overlapping estimates (Fig. 3A–D). Human F1 scores were uniformly high (0.964–0.991 on average; Table 1), accompanied by very low false positive rates (FPR 0.0023–0.0039), and consistently high sensitivity and specificity across classes. This provides a practical human benchmark for contextualizing model performance.

Relative to this benchmark, EpiVLM achieved strong but lower F1 scores (0.849–0.960; highest for staring at 0.960, 95% CI [0.934, 0.980]; Table 1; Fig. 3A), with high sensitivity across semiologies (0.871–0.953; Fig. 3B). The primary gap was an elevated false positive burden compared with clinicians (model FPR 0.054–0.291 vs human 0.003; Table 1), most pronounced for manual automatisms (FPR 0.291, 95% CI [0.172, 0.436]; specificity 0.709, Fig. 3C, D). The specific results and values are presented in Table S7.

### Performance stratified by age group

**Fig. 4:**
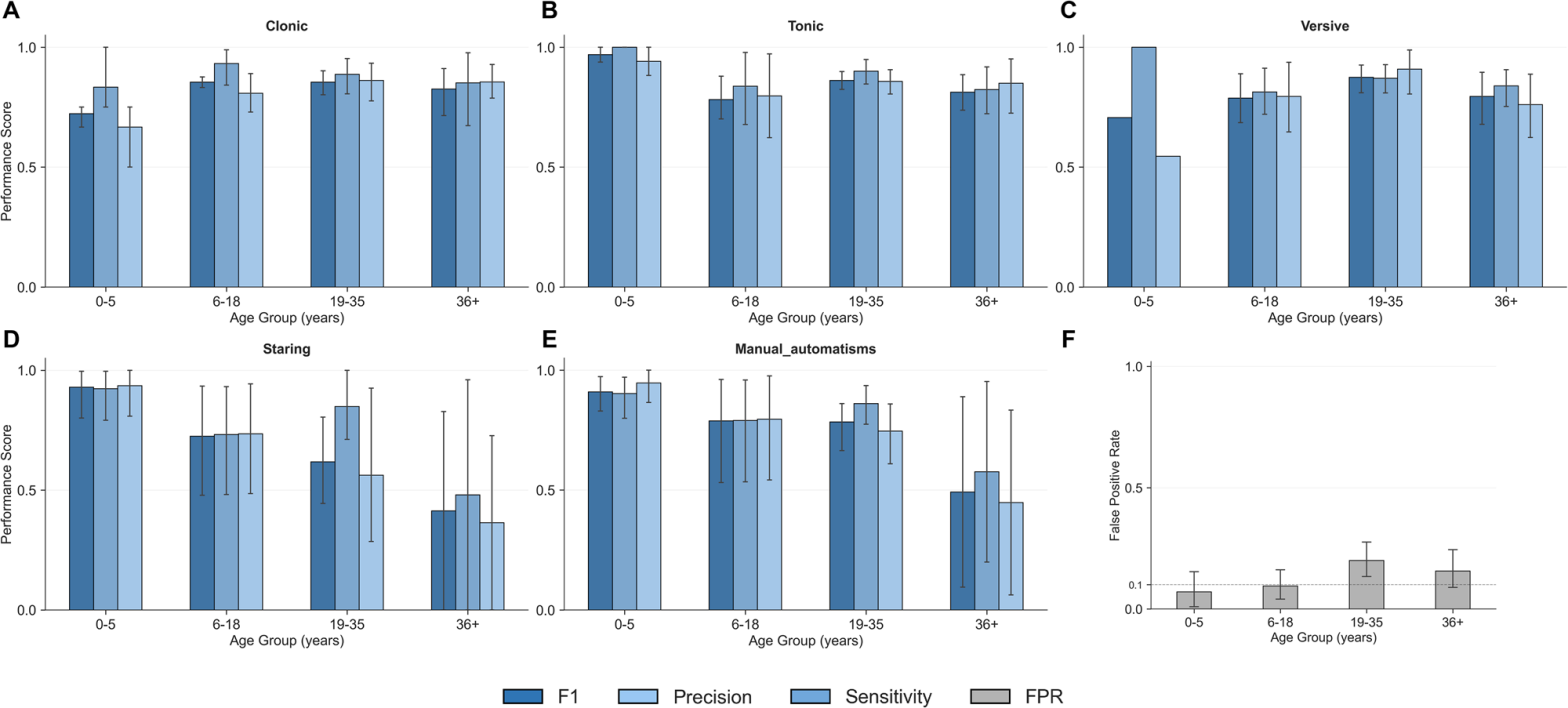
Age-stratified EpiVLM performance across seizure semiology. Model performance is stratified by patient age group (0–5, 6–18, 19–35, and ≥36 years). For each semiology class (**A**, clonic; **B**, tonic; **C**, versive; **D**, staring; **E**, manual automatisms), bars report F1, precision, and sensitivity within each age group. **F** False positive rate (FPR) across age strata. Error bars denote 95% confidence intervals.

We evaluated EpiVLM across four age groups (0–5, 6–18, 19–35, and ≥36 years) (Fig. 4A–E). In the 0–5 years group, recognition was strong for tonic (F1 = 0.969, 95% CI [0.938, 1.000]) and staring (F1 = 0.929, 95% CI [0.801, 0.996]). Clonic performance was initially lower (0–5 years: F1 = 0.722, 95% CI [0.667, 0.750]) but improved for the 6–18 years cohort (F1 = 0.854, 95% CI [0.831, 0.876]). In adults, performance remained stable for clonic (19–35 years: F1 = 0.854, 95% CI [0.801, 0.901]; ≥36 years: F1 = 0.826, 95% CI [0.714, 0.910]) and versive (19–35 years: F1 = 0.874, 95% CI [0.810, 0.925]; ≥36 years: F1 = 0.795, 95% CI [0.678, 0.895]). Conversely, the ≥36 years cohort showed marked reductions with wide confidence intervals for manual automatisms (F1 = 0.492, 95% CI [0.095, 0.889]) and staring (F1 = 0.414, 95% CI [0.000, 0.828]).

Macro-averaged false-positive rates (FPR) were lower in pediatric groups (0–5 years: FPR = 0.064; 6–18 years: FPR = 0.092) than in adults (19–35 years: FPR = 0.207; ≥36 years: FPR = 0.188) (Fig. 4F). By semiology (Figure S2), manual automatisms had the highest adult FPRs (FPR = 0.381–0.414), while versive FPRs remained low (FPR = 0.035–0.056). Estimates for the ≥36 years group warrant cautious interpretation due to smaller sample sizes (Table S6).

### Stepwise prompting and supervised fine-tuning progressively improve semiology recognition

**Fig. 5:**
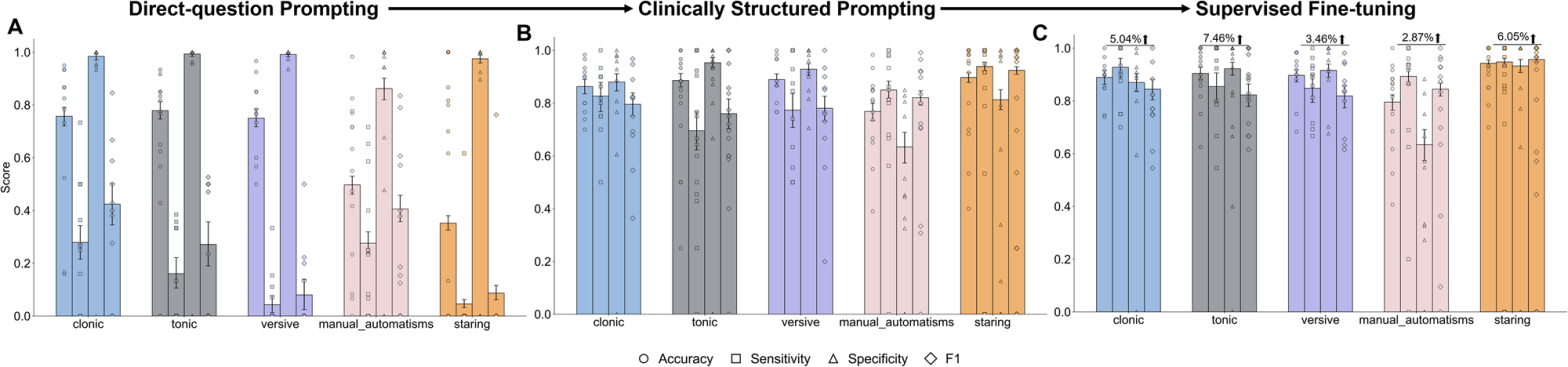
Performance gains from clinical prompting and fine-tuning. Model performance for five seizure semiologies (clonic, tonic, versive, manual automatisms, staring) is shown for three settings: (**A**) direct-question prompting, (**B**) clinically structured prompting, and (**C**) symptom-specific fine-tuning. Bars denote mean across videos; dots indicate individual videos; error bars show 95% bootstrap confidence intervals. Percent labels in (**C**) indicate the mean relative improvement over (**B**), averaged across accuracy, sensitivity, specificity, and F1.

We evaluated three inference regimes across five seizure semiologies to quantify performance contributions from clinical structure and supervised adaptation (Fig. 5A–C). With direct-question prompting, the model remained conservative: specificity was generally high (SPE = 0.862–0.993; FPR = 0.007–0.138), but sensitivity was severely limited. This yielded low overall performance, peaking modestly for clonic (SEN = 0.279, F1 = 0.424) and manual automatisms (SEN = 0.276, F1 = 0.406), while dropping precipitously for tonic, staring, and versive semiologies (SEN = 0.043–0.161, F1 = 0.080–0.271).

Clinically structured prompting—incorporating neurologist-refined descriptors and standardized templates—shifted the model toward more balanced decisions. Accuracy increased to 0.768–0.897, and sensitivity improved across all classes, peaking for staring (SEN = 0.939, 95% CI [0.923, 0.954]) and remaining lowest for tonic (SEN = 0.696, 95% CI [0.623, 0.766]). High specificity was preserved for tonic (SPE = 0.953, 95% CI [0.934, 0.971]) and versive (SPE = 0.929, 95% CI [0.906, 0.949]) classes. However, visually ambiguous semiologies exhibited a clear sensitivity–FPR tradeoff, notably manual automatisms (SPE = 0.634, FPR = 0.366) and staring (SPE = 0.813, FPR = 0.187).

Supervised fine-tuning further strengthened recognition, boosting overall accuracy (Acc=0.795–0.942) and sensitivity (SEN = 0.848–0.947). Sensitivity was highest for staring (SEN = 0.947, 95% CI [0.932, 0.962]) and clonic (SEN = 0.927, 95% CI [0.888, 0.962]) seizures, while manual automatisms, tonic, and versive classes also reached robust levels (SEN = 0.848–0.893). Modest FPR increases were observed for clonic (FPR = 0.130) and versive (FPR = 0.085), whereas the elevated FPR for manual automatisms persisted (FPR = 0.366). Compared with clinically structured prompting, fine-tuning yielded consistent relative improvements across metrics (tonic: 7.46%; staring: 6.05%; clonic: 5.04%; versive: 3.46%; manual automatisms: 2.87%). Full metrics with 95% confidence intervals are reported in Table S4.

### Ablation study on impact of segmentation and fine-tuning

**Fig. 6:**
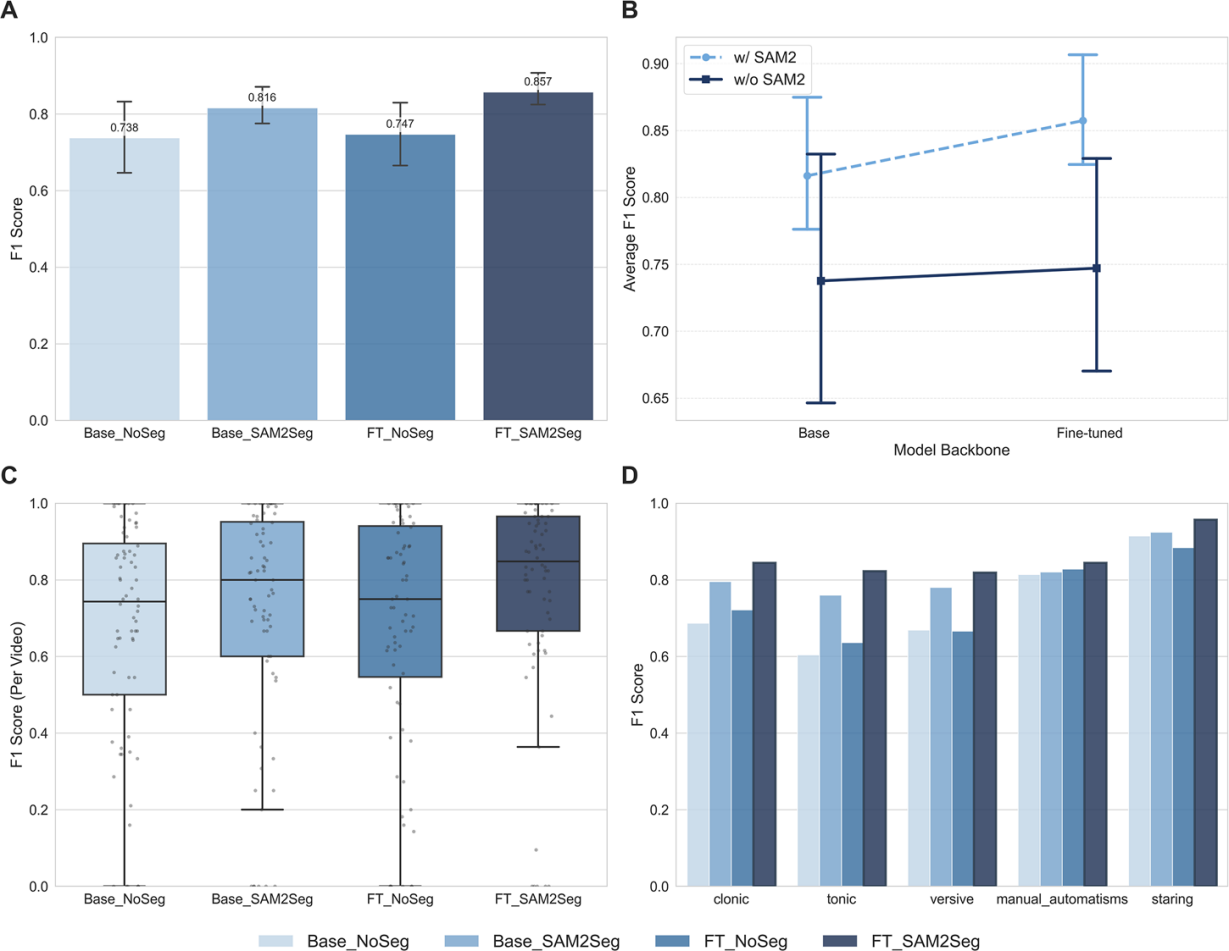
Ablation study on the synergistic impact of segmentation and fine-tuning. **A** Overall comparison of mean F1-scores across four configurations, with the full pipeline (FT_SAM2Seg) achieving the highest performance (0.857). Error bars: 95% CI. **B** Interaction plot demonstrating the additive performance gains from both SAM2-based segmentation and supervised fine-tuning. **C** Box plots of per-video F1 distributions; the narrower interquartile ranges in segmented models (_SAM2Seg) indicate significantly reduced variance and higher stability. **D** Performance stratified by semiology, highlighting that segmentation is critical for static signs (e.g., tonic), while fine-tuning boosts dynamic motor recognition (e.g., clonic).

To isolate the contribution of SAM2 segmentation, we compared the frozen backbone with and without patient masks (Base_SAM2Seg vs. Base_NoSeg). Segmentation increased the mean F1-score (F1 = 0.738 → 0.816) and reduced overall false positives (Fig. 6A, C). The most pronounced gains occurred in tonic seizures, where enhanced specificity yielded a 25.8% relative F1 improvement (F1 = 0.604 → 0.760; SPE = 0.655 → 0.953; FPR = 0.345 → 0.047) (Fig. 6D). Robust improvements were also observed for clonic (F1 = 0.687 → 0.795) and versive (F1 = 0.669 → 0.780) classes. Conversely, gains were marginal for staring and manual automatisms (F1 increases < 0.01), suggesting that residual errors in these classes are less driven by spatial background distraction.

To inject symptom-specific semantics, we further applied supervised fine-tuning (FT_SAM2Seg). This combination yielded additive effects (Fig. 6B), increasing the mean F1-score to 0.857. Fine-tuning consistently boosted performance across all semiologies (+0.024 to +0.063 F1 per class; Table 2). In the fully optimized pipeline, sensitivity peaked for dynamic motor behaviors (clonic SEN = 0.927; manual automatisms SEN = 0.893), while specificity remained robust for tonic (SPE = 0.922) and staring (SPE = 0.932). The comparatively elevated FPR for manual automatisms persisted (FPR = 0.366), indicating an inherent vulnerability to spurious positives.

**Table 2:** Performance impact of segmentation and supervised fine-tuning across five semiologies

Symptom	Method	Accuracy	Sensitivity	F1-Score	Specificity	FPR
Clonic	Base_NoSeg	0.771	0.774	0.687	0.769	0.231
	Base_SAM2Seg	0.863	0.827	0.795	**0.880**	**0.120**
	FT_NoSeg	0.782	0.871	0.721	0.739	0.261
	FT_SAM2Seg	**0.889**	**0.927**	**0.844**	0.870	0.130
Tonic	Base_NoSeg	0.709	0.865	0.604	0.655	0.345
	Base_SAM2Seg	0.886	0.696	0.760	**0.953**	**0.047**
	FT_NoSeg	0.731	**0.917**	0.636	0.667	0.333
	FT_SAM2Seg	**0.904**	0.854	**0.823**	0.922	0.078
Versive	Base_NoSeg	0.776	**0.890**	0.669	0.736	0.264
	Base_SAM2Seg	0.889	0.774	0.780	**0.929**	**0.071**
	FT_NoSeg	0.778	0.872	0.666	0.745	0.255
	FT_SAM2Seg	**0.897**	0.848	**0.819**	0.915	0.085
Manual automatisms	Base_NoSeg	0.761	0.843	0.814	0.625	0.375
	Base_SAM2Seg	0.768	0.850	0.821	0.634	0.366
	FT_NoSeg	0.784	0.836	0.828	0.697	0.303
	FT_SAM2Seg	**0.795**	**0.893**	**0.845**	**0.634**	**0.366**
Staring	Base_NoSeg	0.885	0.923	0.915	0.807	0.193
	Base_SAM2Seg	0.897	0.939	0.924	0.813	0.187
	FT_NoSeg	0.849	0.855	0.884	0.837	0.163
	FT_SAM2Seg	**0.942**	**0.947**	**0.957**	**0.932**	**0.068**

The bold values indicate the best-performing method for each metric within each seizure semiology. Specifically, the highest values are highlighted for Accuracy, Sensitivity, F1-score, and Specificity, whereas the lowest value is highlighted for FPR.

*FT* fine-tuning, *Seg* segmentation, *SAM2* Segment Anything Model 2, *FPR* False Positive Rate.

### High run-to-run stability and low detection latency

**Fig. 7:**
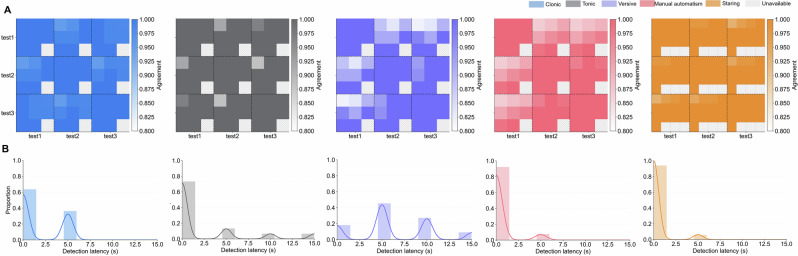
Run-to-run stability and detection latency profiles across seizure types. **A** Heatmaps showing output consistency across three model runs. Each panel corresponds to one seizure symptom. Off-diagonal blocks show pairwise agreement between runs. Each square represents one video; deeper color indicates higher agreement. **B** Distribution of detection latency across videos. Histogram and KDE curve show the proportion of videos by median detection latency. Peaks indicate the latency range where seizures were most consistently detected.

We assessed prediction stability by rerunning inference three times on the same videos and comparing segment-level outputs, then summarizing agreement as the mean per video for each seizure type. Consistency was high across all five semiologies (Fig. 7A).

Staring, tonic, and clonic outputs were effectively invariant across runs, with mean consistency >0.98. Staring showed the highest agreement (0.994; 95% CI [0.986, 1.000]), followed by tonic (0.990; 95% CI [0.971, 1.000]) and clonic (0.987; 95% CI [0.980, 0.994]). Agreement remained strong for manual automatisms (0.954; 95% CI [0.931, 0.975]) and versive seizures (0.949; 95% CI [0.915, 0.978]).

Detection coverage was complete under our operational definition: the model detected every annotated seizure (Fig. 7B). Mean detection latency (mean ± SD) was lowest for manual automatisms (1.68 ± 2.70 s) and staring (0.34 ± 0.71 s), then tonic (1.04 ± 2.72 s), versive (1.95 ± 3.59 s), and clonic (0.61 ± 0.98 s). This ordering tracked sensitivity: semiologies with clearer visual signatures were detected earlier, while more dynamic motor patterns such as manual automatisms and versive tended to show longer delays.

### Robustness across heterogeneous real-world recording conditions

**Fig. 8:**
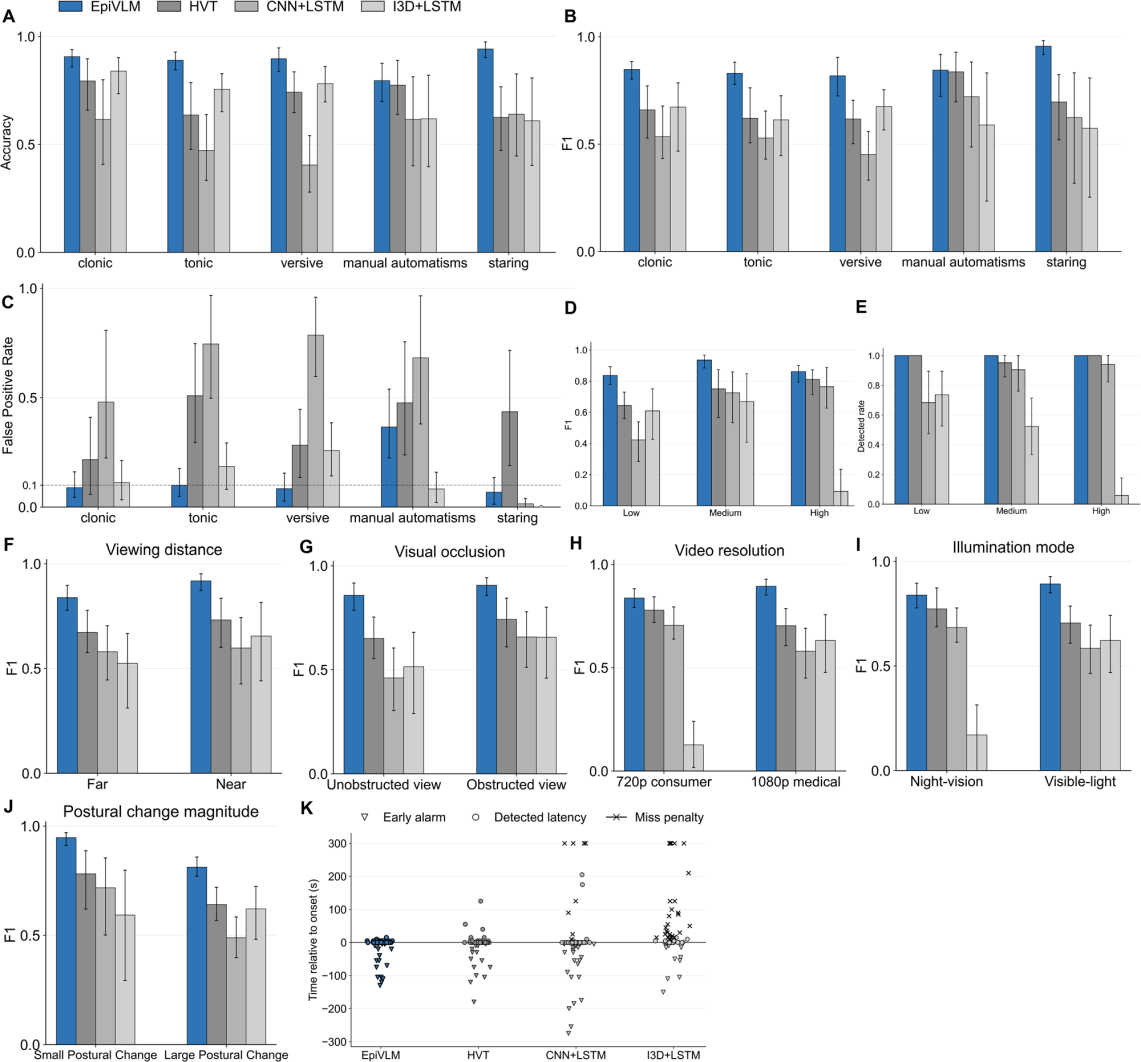
Model performance and real-world robustness. Performance comparison of EpiVLM against three baselines (HVT, CNN + LSTM, I3D + LSTM) on five seizure semiologies and across diverse real-world acquisition conditions. Bars indicate mean performance; error bars denote 95% confidence intervals. **A** Accuracy across five semiologies (clonic, tonic, versive, manual automatisms, staring) for EpiVLM and baselines. **B** F1-score across the same five semiologies. **C** False positive rate across five semiologies. **D** F1-score by recognition difficulty bin (Low 0–1, Medium 2–3, High 4–5; score aggregated from five recording-condition dimensions). **E** Detected rate (≥1 positive clip) by difficulty bin. **F** F1 by viewing distance (Far vs Near). **G** F1 by occlusion (Unobstructed vs Obstructed). **H** F1 by resolution/device class (720p consumer vs 1080p medical). **I** F1 by illumination (Night-vision vs Visible-light). **J** F1 by motion amplitude (Small vs Large postural change). **K** Time-to-onset summary showing early alarms (before onset), detected latency (after onset), and miss penalty (no detection in an entire video).

Across five semiologies, EpiVLM achieved high overall performance (Acc=0.796–0.942; F1 = 0.819–0.956), outperforming three domain-adapted baselines (HVT, I3D + LSTM, CNN + LSTM) on macro-averaged accuracy (Acc = 0.886 vs. 0.550–0.721) and F1-score (F1 = 0.860 vs. 0.572–0.686) (Fig. 8A–B). The largest absolute F1 gains over the best baseline occurred for staring (F1 = 0.956, 95% CI [0.917, 0.982] vs. 0.696) and tonic (F1 = 0.830, 95% CI [0.778, 0.882] vs. 0.621) seizures. Notably, for tonic seizures, EpiVLM balanced precision and recall (PRE = 0.798, REC = 0.865), whereas HVT suffered from low precision despite high recall (PRE = 0.459, REC = 0.959). EpiVLM false-positive rates remained consistently low across clonic, tonic, versive, and staring classes (FPR = 0.068–0.099), while the elevated FPR for manual automatisms (FPR = 0.366, 95% CI [0.224, 0.539]) was consistent with baseline vulnerabilities (e.g., HVT FPR = 0.477) (Fig. 8C).

Under compounding recording challenges (Fig. 8D–E), EpiVLM maintained a 1.00 detection rate across all difficulty bins, yielding robust F1 scores in Low (F1 = 0.835, 95% CI [0.777, 0.893]), Medium (F1 = 0.935, 95% CI [0.884, 0.967]), and High (F1 = 0.861, 95% CI [0.794, 0.902]) strata. In contrast, baselines struggled: HVT maintained high detection rates but lower F1 scores (F1 = 0.644–0.750 in Low/Medium bins), while I3D + LSTM detection rates collapsed precipitously in the High bin (0.059) (Table S8). Regarding time-to-onset dynamics, EpiVLM demonstrated the shortest mean detection latency (1.72 s) compared to baselines (6.23–65.26 s), featuring a tighter latency distribution and fewer missed events (Fig. 8K).

Across individual conditions (Fig. 8F–J), viewing distance and visual occlusion had modest effects on performance (F1 = 0.839–0.918). Large-amplitude motion primarily degraded precision (PRE = 0.953 → 0.766) while sensitivity remained robustly high (SEN = 0.942 → 0.863). More pronounced shifts occurred under challenging visual constraints: 720p consumer cameras and night-vision illumination reduced specificity and elevated FPRs compared to 1080p medical (SPE = 0.688 vs. 0.886; FPR = 0.312 vs. 0.114) and visible-light (FPR = 0.263 vs. 0.119) recordings (Figure S3). To mitigate this, we applied a minimum-duration temporal filter, which successfully improved specificity for 720p (+2.75%) and night-vision (+3.95%) videos without compromising sensitivity (Table S5).

### External validation across domains

**Fig. 9:**
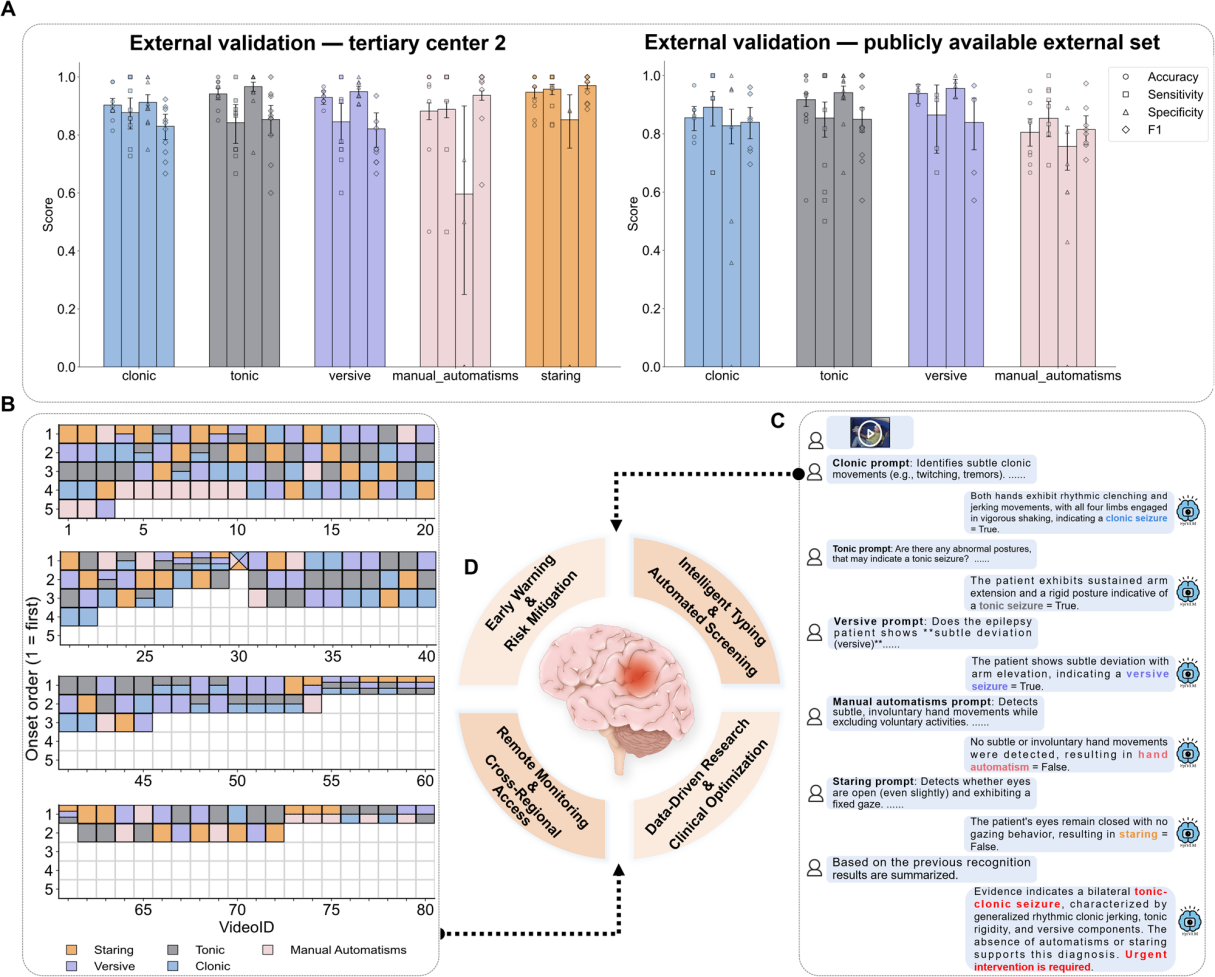
External validation and potential clinical applications. **A** Preliminary external validation on two independent datasets. Bars represent accuracy, sensitivity, specificity, and F1-score for each seizure type, evaluated using the same prompts and thresholds as in the main study. **B** Each row indicates the onset position (1 = first, 5 = last) and each column corresponds to a video. Colored blocks denote detected symptoms, with overlapping segments split visually within the same cell. **C** The model sequentially answers five symptom-specific prompts from a clinical video, returning binary predictions with textual justifications. A final summary integrates the outputs to identify a bilateral tonic–clonic seizure and recommend intervention. **D** Potential clinical applications of EpiVLM. The system enables remote monitoring, automated seizure-type classification, and structured data output. Integration of symptom onset order may support seizure localization and presurgical planning.

We assessed generalizability through dual external validation (Fig. 9A). On the tertiary Center 2 cohort, EpiVLM maintained robust macro performance across five semiologies (Acc = 0.885; SEN = 0.894; F1 = 0.858; SPE = 0.855). Recognition peaked for staring (Acc = 0.942; SEN = 0.947; F1 = 0.957; FPR = 0.068), while tonic and versive classes demonstrated high specificity (SPE = 0.915–0.922; FPR < 0.09). Manual automatisms maintained strong sensitivity (SEN = 0.893) but yielded lower specificity (SPE = 0.634; FPR = 0.366). Performance remained comparably stable on an independent public dataset of four shared semiologies (Acc = 0.879; SEN = 0.866; F1 = 0.836; SPE = 0.871), where versive and tonic seizures again exhibited the highest specificities (SPE > 0.94). Nonparametric tests did not detect statistically significant performance differences across the three centers (Kruskal–Wallis *P* = 0.830) or between the two tertiary cohorts (Mann–Whitney *P* = 0.230).

Specificity under continuous monitoring was evaluated using non-seizure control videos. The video-level false positive rate (FPR) remained consistently low across most phenotypes: tonic (FPR = 0.47%, 95% CI [0.13%, 0.87%]), versive (FPR = 0.66%, 95% CI [0.27%, 1.07%]), clonic (FPR = 1.28%, 95% CI [0.74%, 1.90%]), and staring (FPR = 1.40%, 95% CI [0.83%, 2.01%]). Manual automatisms exhibited a comparatively higher rate (FPR = 2.45%, 95% CI [1.73%, 3.20%]). These estimates indicate reliable false-alarm suppression in varied external environments.

Figure 9C shows a representative multi-symptom inference trace and the resulting clinical interpretation. To characterize temporal evolution of semiology, we analyzed per-video onset sequences and explicitly annotated simultaneous manifestations (Fig. 9B), stratifying videos by the number of observed semiologies. A clear temporal hierarchy emerged: staring or versive movements predominantly appeared first (e.g., 80.0% in 5-symptom videos; 60.0% in 4-symptom videos), frequently progressing to tonic/clonic manifestations (27.7% overall). Conversely, manual automatisms rarely presented as initial features (14.5%). Simultaneous onsets were also highly prevalent (38.6% of overall videos), with tonic–clonic co-onset being the most frequent pairing (12.0%). For automated detection, this hierarchy argues for early sensitivity to behavioral arrest (staring/versive cues) without sacrificing the capacity to recognize concurrent motor presentations.

## Discussion

This study presents EpiVLM, a fine-tuned multimodal vision–language system for automated seizure detection and semiology recognition in hospital and home video. By combining person-centric visual segmentation with task-focused prompting and supervised adaptation, the system achieved low-latency detection and consistent performance across five semiologies in this retrospective cohort. Importantly, EpiVLM is not intended to replace expert interpretation on isolated video clips, but to serve as an adjunctive high-sensitivity screening tool during prolonged monitoring, where continuous manual review is often impractical. In this workflow, the system identifies candidate seizure segments for downstream clinician adjudication, potentially reducing surveillance burden while maintaining expert oversight. Together, the results support the feasibility of AI-assisted video monitoring as an adjunct in settings where continuous expert observation is limited.

Compared with prior video-based approaches, EpiVLM emphasizes clinically grounded inference rather than purely data-driven classification^23,24,26^. The combination of visual segmentation, semiology-specific prompting, and fine-tuning supported recognition across both low-motion and dynamic motor manifestations. Prompt design is particularly relevant in medical AI systems because it can affect reliability and operating characteristics^33,34^, and fine-tuning further improved performance on several challenging semiologies while preserving clinically useful time-to-detection behavior^35^.

The potential applications, conceptually illustrated in Fig. 9D, span inpatient, ambulatory, and low-resource care. In epilepsy monitoring units, earlier automated recognition may help support timely intervention and reduce the risk of prolonged seizures, injury, or status epilepticus^36,37^. In outpatient and home settings, continuous video-based monitoring could improve seizure tracking and support longitudinal treatment assessment^38,39^. More broadly, structured behavioral traces derived from video may also provide useful inputs for future decision-support studies^40,41^.

The high inter-rater agreement observed in this study should be interpreted in light of the annotation task. Unlike de novo diagnostic studies reporting moderate agreement^42^, our raters evaluated predefined semiologies within fixed 5-second windows in videos from confirmed epilepsy cases. Under these constrained conditions, the task is closer to standardized feature identification than open-ended diagnosis, which plausibly reduces variability and is consistent with the high agreement observed here^43^.

Our comparisons with CNN + LSTM, I3D + LSTM, and HVT should be interpreted as system-level benchmarks rather than strictly component-matched comparisons. Because EpiVLM combines clinical prompting, visual segmentation, and supervised adaptation, direct one-to-one comparison with conventional video classifiers is inherently limited. We therefore relied on ablation analyses to clarify the contribution of individual system components.

Despite these promising applications, several methodological and technical limitations merit consideration before large-scale deployment. First, regarding demographic representativeness, epilepsy incidence exhibits a bimodal distribution with a significant burden in older adults^44,45^. Our cohort is enriched for pediatric cases, which limits precision for age-group analyses. Consequently, estimates in the oldest group exhibit wider confidence intervals and must be interpreted cautiously. Expanding recruitment of older adults—who often have different etiologies and comorbidity profiles—will be crucial to strengthen generalizability and characterize performance under continuous monitoring.

Second, evaluating transportability to new environments remains a challenge. While our cross-site evaluation under a fixed training protocol robustly tested generalization to external datasets, it does not fully substitute for leave-one-center-out (LOCO) retraining^46^. A full LOCO evaluation was deferred here because current multi-center sample sizes remain unbalanced, which would yield unstable model training in certain folds. However, as external data from collaborating institutions continue to accrue, performing strict LOCO evaluations will be a crucial next step.

Third, specificity declined under lower-quality visual conditions, particularly reduced resolution and night vision, which may increase false alarms and reduce trust in home monitoring^25^. Although a minimum-duration temporal filter improved specificity^47^, future work should further address artifact-prone conditions through better temporal aggregation, ambiguity-aware prompting, and multimodal fusion with complementary physiological signals such as EEG, accelerometry, or heart rate^48,49^.

In summary, EpiVLM advances toward practical, cross-environment seizure video monitoring by integrating spatial focus, clinically grounded prompting, and supervised adaptation. While broader prospective and multi-center validation is needed—including LOCO evaluation and improved age representativeness—the system provides a foundation for remote monitoring, workflow integration, and outcome-linked evaluation in epilepsy care.

## Methods

### Ethics approvals, video cohorts, and expert annotation reliability

This study was approved by the Medical Ethics Committee of West China Hospital of Sichuan University (2023(1271)) and Ethics Committee of Xiamen Humanity Hospital (HAXM-MEC-20250217-009-01), and has been registered in the Chinese Clinical Trial Registry (ChiCTR2400081104 | |Registration Date:2024.02.22). Video recordings of patients with epilepsy were captured using cameras placed at the bedside in the epilepsy monitoring units (EMUs) of both institutions, as well as in the home environments of consenting participants. Informed consent to participate in the study was obtained from all participants or their parent/legal guardian in the case of children under the age of 16.

During preprocessing, videos with significant occlusion, poor visibility, or lacking a clear view of the patient were excluded. After quality control, a total of 232 usable videos were retained: 174 hospital recordings (118 from Xiamen Humanity Hospital (tertiary center 1); 56 from West China Hospital (tertiary center 2)), 38 home recordings, and 20 recordings from an open-access public dataset^30^. These videos encompassed both seizure and non-seizure periods of daily activity and captured five major seizure semiologies: tonic, clonic, manual automatisms, staring, and versive. The final dataset included 127 unique patients (median age 16.29 years [IQR 20.7; Q1–Q3, 5.3–26.0]; range 2–55 years; 69 male, 58 female). Specifically, the cohort was partitioned into subsets of 43 patients for fine-tuning/training, 28 for internal testing, and 56 for external validation. Detailed demographic and clinical information is provided in Table S1.

To ensure annotation accuracy, all videos were independently reviewed and labeled by two neurologists with clinical expertise in epilepsy, following standardized annotation protocols. Inter-rater reliability was assessed using all annotated videos with Cohen’s *κ* statistics^50^, and 95% confidence intervals were estimated by bootstrapping. Discrepancies between annotators were adjudicated by a senior neurologist, and the consensus annotations were used as the ground truth for subsequent model training and evaluation. In addition, we quantified each rater’s performance against the adjudicated reference labels and compared this human benchmark with the model’s performance.

### Video preprocessing: standardization, patient masking, and 5-s segmentation

Videos were partitioned into non-overlapping 5-second windows (stride = 5 s; timestamps defined at the window start) aligned with the clinical annotation timeline. For each window, we generated a 10-frame stack at 2 fps for frame-based inference and extracted patient silhouettes using Segment Anything Model 2 (SAM2)^31^; the resulting masks were applied to both the 10-frame stacks and the corresponding native-rate 5-second clips. Inference used the 10-frame stack for staring seizures, and the original-rate 5-s clip for clonic, tonic, versive, and manual automatisms to preserve within-window temporal dynamics. The same preprocessing and masking were used across all compared methods.

### Vision–language backbone and clinically structured prompting

For automated seizure recognition, we employed the Qwen2.5-VL-32B multimodal model^32^ as the vision–language backbone. The model received 5-second video inputs generated during preprocessing together with semiology-specific text prompts, and produced binary predictions for the presence or absence of each target seizure semiology.

Prompt design was refined through prompt engineering^34,51–53^ to standardize inference across the five semiologies (clonic, tonic, versive, manual automatisms, and staring). The final prompts incorporated neurologist-refined semiologic descriptors, explicit task instructions, and standardized response templates. When applicable, prior inference context was incorporated to improve temporal consistency and reduce spurious positives. Direct-question prompts are listed in Table S2, and representative optimized prompt templates are provided in Table S3.

### Supervised fine-tuning and training configuration

We fine-tuned Qwen2.5-VL-32B using segment-level targets derived from the adjudicated annotations. For each segment, neurologists defined the expected model response, including the presence or absence of symptoms and their semiology type.

To prevent data leakage, all dataset splits were performed at the patient level. Data from Center 1 and home recordings were used for model development and internal testing, with strict patient-level separation between the fine-tuning and internal-test subsets. Data from Center 2 and the public dataset were reserved exclusively for external validation, ensuring no patient overlap between development and evaluation. The overall data workflow is shown in Figure S1.

After quality control and consensus reconciliation by two neurologists, the final fine-tuning dataset comprised 491 clonic samples (304 positive, 187 negative), 540 tonic samples (359 positive, 181 negative), 266 versive samples (127 positive, 139 negative), 215 manual automatisms samples (132 positive, 83 negative), and 355 staring samples (190 positive, 165 negative).

Data were split into training and test sets at an 8:2 ratio, and 20% of the training set was further reserved as a validation set for early stopping based on validation loss. During development, the fine-tuning dataset was iteratively refined by removing noise and adjusting the positive-to-negative ratio based on model evaluation. Fine-tuning was implemented using the LLaMA-Factory framework^54^ with QLoRA (Quantized Low-Rank Adaptation)^55^ for parameter-efficient adaptation. All computations were performed on an Ubuntu system with three NVIDIA A100-PCIE-40GB GPUs.

### Ablation study design and component analysis

To disentangle the specific contributions of spatial attention and domain-specific adaptation, we implemented a structured ablation study using a 2 × 2 factorial design. We established an absolute baseline (Base_NoSeg) using the pre-trained Qwen2.5-VL-32B model for zero-shot inference on raw, unmasked frames. This was compared against two isolated-component variants: Base_SAM2Seg, which applied SAM2-based masking to the pre-trained model to quantify the impact of background noise mitigation, and FT_NoSeg, which utilized the fine-tuned model on raw frames to evaluate supervised domain adaptation independent of spatial filtering. These configurations culminated in the proposed fully integrated pipeline (FT_SAM2Seg), combining both strategies. All variants were evaluated on the identical test set and metrics to ensure direct comparability.

### Baseline models and standardized evaluation protocol

We benchmarked EpiVLM against three open-source baselines (HVT^56^, CNN + LSTM^24^, and I3D + LSTM^57^) under a strictly harmonized evaluation protocol. All models were trained on the same training split and evaluated on the same test split. Videos were segmented into identical non-overlapping 5-s windows, and an identical pre-processing pipeline (including standardization and masking) was applied uniformly across methods. Under this locked setup, segment-level classification and video-level event detection outcomes are directly comparable across models. For videos containing multiple semiologies, we further derived the onset order of semiologies to enable standardized summaries of within-video semiology progression.

### Robustness analysis across heterogeneous recording conditions

To characterize robustness under real-world recording heterogeneity, we annotated each test video along five predefined condition dimensions and performed stratified evaluation using the same fixed thresholds as in the primary analysis. (1) Viewing distance (Far vs Near). Distance was operationalized by the patient’s field-of-view. Videos in which the patient’s full body was visible for the majority of the recording were labeled Far; videos showing only the upper body (approximately waist/chest and above) were labeled Near. (2) Illumination mode (Night-vision vs Visible-light). Illumination was determined by the imaging mode. Visible-light videos exhibited normal color information, whereas Night-vision videos were monochrome (black/white/gray), consistent with infrared night-vision recording. (3) Visual occlusion (Unobstructed vs Obstructed). Occlusion was defined by whether the patient’s motor-relevant body regions were visually blocked. Videos were labeled Obstructed if any object (e.g., blanket) covered or blocked the patient below the chest for the majority of the recording; otherwise, they were labeled Unobstructed. (4) Motion amplitude (Small vs Large postural change). Motion amplitude was categorized based on the overall magnitude of posture change during the seizure period. Large indicated prominent whole-body or trunk displacement/postural transition (e.g., major body repositioning), whereas Small indicated limited postural change with movements largely confined to local body parts. (5) Device class/resolution (720p consumer vs 1080p medical). The device class was defined by acquisition source and nominal resolution. Hospital videos were recorded by a standardized VEEG camera system at 1920 × 1080 (1080p medical), whereas home videos were recorded using consumer-grade cameras and were typically 1280 × 720 (720p consumer).

Based on the five recording-condition dimensions, we constructed a composite recognition-difficulty score for each video. For each dimension, the presence of the more challenging condition was coded as 1 and the less challenging condition as 0 (Distance: Far = 1, Near = 0; Occlusion: Obstructed = 1, Unobstructed = 0; Device class: 720p consumer = 1, 1080p medical = 0; Illumination: Night-vision = 1, Visible-light = 0; Motion amplitude: Large postural change = 0, Small = 1). The overall difficulty score was defined as the sum of these five binary indicators (range 0–5). Videos were then categorized into Low (0–1), Medium (2–3), and High (4–5) difficulty bins for stratified analyses. All recording-condition labels were assigned at the video level by two independent annotators using the prespecified criteria above. Disagreements were resolved by consensus during a joint review (with reference to the full video context), yielding a single adjudicated label per dimension for each video. For lower-quality recordings, such as 720p consumer videos and night-vision, a minimum-duration temporal filter was applied to the segment-level predictions to reduce spurious detections while preserving sensitivity. These labels were then used for stratified evaluation under the same preprocessing, windowing, and fixed thresholds as the primary analysis.

### External datasets and evaluation procedures

Generalizability was assessed using two independent external datasets: (i) the Tertiary Epilepsy Center 2 dataset (56 videos; no overlap in patients or videos), annotated under the same standardized protocol and adjudication; and (ii) a publicly available dataset (20 videos)^30^ annotated using our study protocol. We applied the same previously fine-tuned model to both cohorts with fixed weights and no retraining, recalibration, or additional tuning. For both datasets, inference used the identical 5-s windowing, preprocessing, masking pipeline, and optimized prompts as in the primary analysis.

### Outcome definitions, stability metrics and detection latency

We report Accuracy, Precision, Sensitivity (Recall), Specificity, False Positive Rate (FPR) and F1-score. Unless otherwise specified, all classification metrics were computed at the segment level, with each non-overlapping 5-s window treated as one evaluation unit; accordingly, FPR was defined as

$${FPR}=\frac{{FP}}{{FP}+{TN}}$$

where$\,{FP}$ and$\,{TN}$ denote falsely predicted positive and correctly predicted negative segments among all ground-truth negative segments, respectively. Run-to-run stability was evaluated using three independent inference runs with identical splits, prompts, and fixed thresholds, summarized as the mean pairwise agreement across runs; 95% confidence intervals were obtained via patient-level bootstrap resampling (1,000 iterations). Detection latency was defined as the time from annotated onset to the first positive 5-s window. We additionally report detected rate (≥1 detection after onset), early-alarm rate (first detection before onset), and a miss penalty for videos with no detection.

## Supplementary information

Supplementary information

## Data Availability

The video dataset used in this study contains sensitive patient information and cannot be made publicly available due to ethical and privacy concerns. However, researchers with legitimate and appropriate academic purposes may request access to the data by contacting the corresponding author (LC) via leilei_25@126.com.

## Code availability

The code for the EpiVLM system is available[https://github.com/Michael98CN/EpiVLMs].Experiments conducted on Python 3.10.16, PyTorch 2.5.1, Transformers 4.51.3, and three NVIDIA A100-PCIE-40GB GPUs running Ubuntu 20.04.6.
