# Supplementary material for: Towards generalizable seizure monitoring: EpiVLM for cross-environment detection and classification
Source: NPJ Digit Med. 2026 May 26;9:635. doi: 10.1038/s41746-026-02810-3 (PMC13482623; doi:10.1038/s41746-026-02810-3)
Supplement: Supplementary file 1 [file 41746_2026_2810_MOESM1_ESM.pdf]

Table S1.Baseline demographic, antiseizure medication, and seizure video characteristics in patients with epilepsy

VideoID	PatientID	Age(year)	Gender	Duration_s	ASMs_use_type	Tonic	Clonic	Versive	Manual_automatisms	Staring	Video_type	Split	Distance	Illumination	Occlusion	MotionAmplitude	DeviceClass
26432_CMVideo00007	26432	5	F	300	0	0	0	0	0	1	Hospital	Train	Far	Visible-light	Unobstructed	Small	Medical-1080p
26432_CMVideo00008	26432	5	F	300	0	0	0	0	0	1	Hospital	Train	Far	Visible-light	Unobstructed	Small	Medical-1080p
27139_CMVideo00120C	27139	2.42	F	258	1	0	0	0	1	0	Hospital	Test	Far	Visible-light	Unobstructed	Large	Medical-1080p
27139_CMVideo00121	27139	2.42	F	300	1	0	0	0	1	1	Hospital	Test	Far	Visible-light	Unobstructed	Large	Medical-1080p
27139_CMVideo00122	27139	2.42	F	300	1	1	0	0	0	1	Hospital	Test	Far	Visible-light	Unobstructed	Small	Medical-1080p
27139_CMVideo00123A	27139	2.42	F	296	1	1	0	0	1	0	Hospital	Test	Far	Visible-light	Unobstructed	Large	Medical-1080p
27246_CMVideo00048	27246	19	F	300	5	1	1	1	0	0	Hospital	Train	Far	Visible-light	Obstructed	Large	Medical-1080p
27275_CMVideo00004	27275	15	M	300	2	0	0	1	1	0	Hospital	Test	Near	Visible-light	Obstructed	Small	Medical-1080p
27283_CMVideo00856	27283	33	M	300	3	0	0	1	1	0	Hospital	Train	Far	Visible-light	Unobstructed	Small	Medical-1080p
27283_CMVideo00857	27283	33	M	300	3	0	0	1	0	0	Hospital	Train	Far	Visible-light	Unobstructed	Small	Medical-1080p
26318_CMVideo00179	26318	54	M	300	2	0	0	0	0	1	Hospital	Train	Near	Visible-light	Unobstructed	Small	Medical-1080p
26325_CMVideo00028	26325	27	M	300	2	1	1	0	0	0	Hospita	Train	Far	Visible-l	Obstruct	Large	Medical-1

											l			ight	ed		080p
26325_CMVideo00029	26325	27	M	300	2	1	1	1	0	0	Hospital	Train	Far	Visible-light	Obstructed	Large	Medical-1080p
26334_CMVideo00161	26334	3	M	300	1	0	1	0	0	0	Hospital	Train	Near	Visible-light	Obstructed	Small	Medical-1080p
26334_CMVideo00190C	26334	3	M	296	1	0	1	0	0	0	Hospital	Train	Near	Visible-light	Obstructed	Small	Medical-1080p
26350_CMVideo00005	26350	5	M	300	3	0	0	1	0	0	Hospital	Train	Near	Visible-light	Obstructed	Small	Medical-1080p
26350_CMVideo00049	26350	5	M	300	3	0	0	0	0	1	Hospital	Train	Near	Visible-light	Obstructed	Small	Medical-1080p
26350_CMVideo00050A	26350	5	M	297	3	0	0	0	0	1	Hospital	Train	Near	Visible-light	Obstructed	Small	Medical-1080p
26360_CMVideo00098	26360	4	M	300	2	0	0	0	0	1	Hospital	Train	Far	Visible-light	Unobstructed	Small	Medical-1080p
25691_CMVideo00006	25691	2.33	F	300	4	0	0	0	1	1	Hospital	Test	Near	Visible-light	Obstructed	Small	Medical-1080p
25691_CMVideo00053	25691	2.33	F	300	4	0	0	0	1	1	Hospital	Test	Near	Visible-light	Obstructed	Small	Medical-1080p
26730_CMVideo00031	26730	7	F	300	3	0	0	0	0	1	Hospital	Train	Far	Visible-light	Unobstructed	Small	Medical-1080p
26730_CMVideo00185	26730	7	F	300	3	1	0	1	0	1	Hospital	Train	Far	Visible-light	Obstructed	Large	Medical-1080p
26328_CMVideo00021	26328	3	F	300	3	1	1	0	0	0	Hospital	Train	Near	Visible-light	Obstructed	Large	Medical-1080p
26328_CMVideo00029	26328	3	F	300	3	1	1	0	0	0	Hospita	Train	Near	Visible-l	Obstruct	Large	Medical-1

											l			ight	ed		080p
26328_CMVideo00047	26328	3	F	300	3	1	1	0	0	1	Hospital	Train	Near	Visible-light	Obstructed	Large	Medical-1080p
26328_CMVideo00139	26328	3	F	300	3	0	1	0	0	1	Hospital	Train	Near	Visible-light	Obstructed	Small	Medical-1080p
25707_CMVideo00021	25707	14	F	300	5	1	0	0	0	0	Hospital	Train	Far	Visible-light	Obstructed	Small	Medical-1080p
26544_CMVideo00090	26544	15	F	300	5	1	1	1	0	0	Hospital	Test	Near	Visible-light	Obstructed	Large	Medical-1080p
26544_CMVideo00091	26544	15	F	300	5	1	1	0	0	0	Hospital	Test	Near	Visible-light	Unobstructed	Large	Medical-1080p
26560_CMVideo00086	26560	29	M	300	2	0	0	0	1	0	Hospital	Train	Far	Visible-light	Unobstructed	Small	Medical-1080p
26476_CMVideo00026	26476	14	F	300	5	1	0	0	0	1	Hospital	Train	Far	Visible-light	Obstructed	Small	Medical-1080p
26511_CMVideo00033	26511	30	M	300	2	1	1	1	0	0	Hospital	Test	Far	Visible-light	Obstructed	Large	Medical-1080p
26719_CMVideo00096	26719	10	F	300	1	1	1	1	0	0	Hospital	Test	Near	Visible-light	Obstructed	Large	Medical-1080p
26951_CMVideo00297	26951	36	F	300	3	1	1	1	0	0	Hospital	Test	Far	Visible-light	Unobstructed	Large	Medical-1080p
26951_CMVideo00383C	26951	36	F	198	3	1	1	1	1	0	Hospital	Test	Far	Visible-light	Obstructed	Large	Medical-1080p
26628_CMVideo00264	26628	28	M	300	3	1	1	1	0	0	Hospital	Test	Far	Visible-light	Unobstructed	Large	Medical-1080p
25820_CMVideo00011	25820	3.33	F	300	4	0	0	1	0	0	Hospita	Train	Near	Visible-l	Obstruct	Small	Medical-1

											l			ight	ed		080p
25701_CMVideo00038	25701	8	M	300	3	0	0	0	1	1	Hospital	Test	Near	Visible-light	Obstructed	Small	Medical-1080p
27618_CMVideo00458	27618	10.42	F	300	2	1	1	1	0	1	Hospital	Train	Far	Visible-light	Unobstructed	Large	Medical-1080p
27505_CMVideo00473	27505	26	M	300	3	1	1	0	0	1	Hospital	Train	Far	Visible-light	Obstructed	Large	Medical-1080p
27228_CMVideo00152	27228	11	M	300	4	1	0	1	0	1	Hospital	Train	Far	Visible-light	Unobstructed	Large	Medical-1080p
27190_CMVideo00048	27190	6.33	M	300	1	1	1	1	0	1	Hospital	Train	Far	Visible-light	Unobstructed	Large	Medical-1080p
27179_CMVideo00189	27179	11.5	M	300	3	1	1	1	0	1	Hospital	Test	Far	Visible-light	Unobstructed	Large	Medical-1080p
27109_CMVideo00108	27109	35	F	300	3	1	1	1	0	1	Hospital	Test	Far	Visible-light	Obstructed	Large	Medical-1080p
26887_CMVideo00006	26887	4.33	F	300	3	0	0	0	1	0	Hospital	Test	Far	Visible-light	Obstructed	Small	Medical-1080p
26474_CMVideo00168	26474	24	M	300	2	1	1	1	1	1	Hospital	Test	Near	Visible-light	Obstructed	Large	Medical-1080p
26699_CMVideo00415	26699	33	M	300	2	1	1	1	0	1	Hospital	Train	Far	Visible-light	Unobstructed	Large	Medical-1080p
26527_CMVideo00181	26527	50	F	300	3	1	1	0	1	1	Hospital	Train	Near	Visible-light	Unobstructed	Large	Medical-1080p
26945_CMVideo00007	26945	3	F	300	4	1	0	0	0	1	Hospital	Train	Near	Night-vision	Obstructed	Small	Medical-1080p
25694_CMVideo00056	25694	42	M	300	3	0	1	0	0	0	Hospita	Train	Far	Visible-l	Unobstr	Small	Medical-1

											l			ight	ucted		080p
25694_CMVideo00056_1	25694	42	M	300	3	0	1	0	0	0	Hospital	Train	Near	Visible-light	Unobstructed	Small	Medical-1080p
25694_CMVideo00057	25694	42	M	300	3	0	1	0	0	0	Hospital	Train	Near	Visible-light	Unobstructed	Small	Medical-1080p
25696_CMVideo00045	25696	10	M	300	2	0	0	1	1	1	Hospital	Train	Far	Visible-light	Obstructed	Small	Medical-1080p
25696_CMVideo00045_1	25696	10	M	300	2	1	0	1	1	0	Hospital	Train	Near	Visible-light	Obstructed	Small	Medical-1080p
25696_CMVideo00046	25696	10	M	300	2	1	0	1	1	0	Hospital	Train	Far	Visible-light	Obstructed	Large	Medical-1080p
25702_CMVideo00093	25702	27	M	300	3	1	1	1	1	1	Hospital	Test	Far	Visible-light	Unobstructed	Large	Medical-1080p
25702_CMVideo00093_1	25702	27	M	300	3	1	1	1	1	1	Hospital	Test	Near	Visible-light	Obstructed	Large	Medical-1080p
25702_CMVideo00094	25702	27	M	300	3	1	1	1	1	0	Hospital	Test	Far	Visible-light	Unobstructed	Large	Medical-1080p
25702_CMVideo00094_1	25702	27	M	300	3	1	1	1	1	1	Hospital	Test	Near	Visible-light	Unobstructed	Large	Medical-1080p
25712_CMVideo00026	25712	5	F	300	2	0	0	0	1	0	Hospital	Train	Far	Visible-light	Unobstructed	Small	Medical-1080p
25720_CMVideo00049	25720	6	F	300	4	0	0	0	0	1	Hospital	Train	Far	Visible-light	Unobstructed	Small	Medical-1080p
25726_CMVideo00076	25726	3.67	F	300	2	1	1	0	0	1	Hospital	Test	Near	Visible-light	Unobstructed	Large	Medical-1080p
25726_CMVideo00076_1	25726	3.67	F	300	2	1	1	0	0	0	Hospita	Test	Far	Visible-l	Obstruct	Large	Medical-1

											l			ight	ed		080p
25798_CMVideo00286	25798	7.42	M	300	4	1	0	1	0	1	Hospital	Train	Near	Visible-light	Obstructed	Large	Medical-1080p
25798_CMVideo00286_1	25798	7.42	M	300	4	1	0	1	0	0	Hospital	Train	Far	Visible-light	Obstructed	Large	Medical-1080p
25798_CMVideo00381_1	25798	7.42	M	300	4	0	0	1	0	0	Hospital	Train	Near	Visible-light	Obstructed	Large	Medical-1080p
25798_CMVideo00381	25798	7.42	M	300	4	1	0	1	0	0	Hospital	Train	Near	Visible-light	Unobstructed	Small	Medical-1080p
26550_CMVideo00210	26550	3	F	300	2	1	0	0	0	0	Hospital	Train	Near	Visible-light	Obstructed	Small	Medical-1080p
26550_CMVideo00192	26550	3	F	300	2	1	0	0	0	0	Hospital	Train	Near	Visible-light	Obstructed	Small	Medical-1080p
26550_CMVideo00147	26550	3	F	300	2	1	0	1	0	1	Hospital	Train	Near	Visible-light	Obstructed	Small	Medical-1080p
26550_CMVideo00147_1	26550	3	F	300	2	1	0	1	0	0	Hospital	Train	Far	Visible-light	Obstructed	Small	Medical-1080p
26550_CMVideo00192_1	26550	3	F	300	2	1	0	0	0	0	Hospital	Train	Far	Visible-light	Obstructed	Small	Medical-1080p
26550_CMVideo00197	26550	3	F	300	2	1	1	1	0	1	Hospital	Train	Near	Visible-light	Obstructed	Large	Medical-1080p
26550_CMVideo00197_1	26550	3	F	300	2	1	1	1	0	0	Hospital	Train	Far	Visible-light	Obstructed	Large	Medical-1080p
26550_CMVideo00198	26550	3	F	300	2	1	1	1	0	0	Hospital	Train	Far	Visible-light	Unobstructed	Large	Medical-1080p
26550_CMVideo00198_1	26550	3	F	250	2	1	1	1	0	0	Hospita	Train	Far	Visible-l	Unobstr	Large	Medical-1

											l			ight	ucted		080p
26550_CMVideo00210_1	26550	3	F	300	2	1	0	1	0	0	Hospital	Train	Far	Visible-light	Obstructed	Small	Medical-1080p
26593_CMVideo00051	26593	18	M	300	2	1	1	1	0	1	Hospital	Train	Far	Visible-light	Unobstructed	Large	Medical-1080p
26637_CMVideo00214	26637	6	F	300	2	1	1	1	0	1	Hospital	Train	Near	Visible-light	Unobstructed	Large	Medical-1080p
26637_CMVideo00215A	26637	6	F	252	2	1	1	1	0	1	Hospital	Train	Far	Visible-light	Unobstructed	Large	Medical-1080p
26637_CMVideo00042	26637	6	F	300	2	1	1	0	0	1	Hospital	Train	Near	Visible-light	Unobstructed	Large	Medical-1080p
26637_CMVideo00108	26637	6	F	300	2	1	1	1	0	1	Hospital	Train	Near	Visible-light	Unobstructed	Large	Medical-1080p
26637_CMVideo00191	26637	6	F	300	2	1	1	1	1	1	Hospital	Train	Near	Visible-light	Unobstructed	Large	Medical-1080p
26637_CMVideo00042_1	26637	6	F	300	2	1	1	0	0	1	Hospital	Train	Far	Visible-light	Unobstructed	Large	Medical-1080p
26637_CMVideo00108_1	26637	6	F	300	2	1	1	1	0	0	Hospital	Train	Far	Visible-light	Unobstructed	Large	Medical-1080p
26637_CMVideo00192	26637	6	F	300	2	1	1	1	1	1	Hospital	Train	Far	Visible-light	Unobstructed	Large	Medical-1080p
26637_CMVideo00216A	26637	6	F	280	2	1	1	1	0	0	Hospital	Train	Far	Visible-light	Unobstructed	Large	Medical-1080p
26637_CMVideo00214C	26637	6	F	37	2	0	0	0	1	0	Hospital	Train	Far	Visible-light	Unobstructed	Large	Medical-1080p
26652_CMVideo00092	26652	13	F	300	2	1	1	1	0	1	Hospita	Test	Near	Visible-l	Obstruct	Large	Medical-1

											l			ight	ed		080p
26686_CMVideo00072	26686	16	M	300	2	1	1	1	0	1	Hospital	Train	Near	Visible-light	Unobstructed	Large	Medical-1080p
26707_CMVideo00019	26707	13	M	300	4	1	0	0	0	0	Hospital	Train	Near	Visible-light	Unobstructed	Small	Medical-1080p
26718_CMVideo00008	26718	18	M	300	1	1	0	0	1	0	Hospital	Train	Near	Visible-light	Obstructed	Small	Medical-1080p
26778_CMVideo00019	26778	3.75	M	300	3	1	0	1	0	1	Hospital	Test	Near	Visible-light	Unobstructed	Small	Medical-1080p
26808_CMVideo00224	26808	6	M	300	3	1	0	1	0	1	Hospital	Train	Near	Visible-light	Unobstructed	Large	Medical-1080p
26873_CMVideo00026	26873	9	M	300	1	1	0	1	1	0	Hospital	Test	Far	Visible-light	Unobstructed	Large	Medical-1080p
26873_CMVideo00138	26873	9	M	300	1	1	0	0	1	0	Hospital	Test	Near	Visible-light	Obstructed	Small	Medical-1080p
27099_CMVideo00043	27099	10.75	M	300	2	0	0	0	1	0	Hospital	Train	Far	Visible-light	Unobstructed	Small	Medical-1080p
27253_CMVideo00099	27253	11.83	F	300	4	1	0	0	0	0	Hospital	Train	Near	Visible-light	Obstructed	Small	Medical-1080p
27260_CMVideo00013	27260	9.33	M	300	2	1	1	1	1	1	Hospital	Train	Near	Visible-light	Obstructed	Large	Medical-1080p
27499_CMVideo00159	27499	10.08	M	300	1	0	0	0	0	1	Hospital	Test	Near	Visible-light	Unobstructed	Small	Medical-1080p
27499_CMVideo00159_1	27499	10.08	M	300	1	0	0	0	0	1	Hospital	Test	Far	Visible-light	Unobstructed	Small	Medical-1080p
25698_CMVideo00208	25698	14	M	300	2	1	1	1	0	1	Hospita	Train	Near	Visible-l	Obstruct	Large	Medical-1

											l			ight	ed		080p
25700_CMVideo00006	25700	13	M	300	3	0	0	0	0	1	Hospital	Test	Near	Visible-light	Obstructed	Small	Medical-1080p
25700_CMVideo00007	25700	13	M	300	3	0	0	0	0	1	Hospital	Test	Near	Visible-light	Obstructed	Small	Medical-1080p
25700_CMVideo00197	25700	13	M	300	3	0	0	0	0	1	Hospital	Test	Near	Visible-light	Obstructed	Small	Medical-1080p
25700_CMVideo00198	25700	13	M	300	3	0	0	0	0	1	Hospital	Test	Near	Visible-light	Obstructed	Small	Medical-1080p
25716_CMVideo00192A	25716	6	F	60	4	0	0	0	0	1	Hospital	Train	Near	Visible-light	Obstructed	Small	Medical-1080p
25802_CMVideo00094	25802	2.92	F	300	4	0	1	0	0	0	Hospital	Train	Near	Visible-light	Obstructed	Small	Medical-1080p
25859_CMVideo00035	25859	2.92	F	300	5	0	0	0	0	1	Hospital	Test	Near	Visible-light	Obstructed	Small	Medical-1080p
25859_CMVideo00036	25859	2.92	F	300	5	0	0	0	0	1	Hospital	Test	Near	Visible-light	Obstructed	Small	Medical-1080p
26213_CMVideo00029	26213	3	F	300	1	0	0	0	0	1	Hospital	Test	Near	Visible-light	Unobstructed	Small	Medical-1080p
26824_CMVideo00150	26824	10.5	F	300	3	0	0	0	0	0	Hospital	Test	Near	Visible-light	Obstructed	Small	Medical-1080p
26864_CMVideo00142	26864	19	M	300	0	0	0	0	0	0	Hospital	Train	Near	Visible-light	Obstructed	Small	Medical-1080p
26951_CMVideo00139C	26951	36	F	126	3	0	0	0	0	0	Hospital	Test	Near	Visible-light	Obstructed	Small	Medical-1080p
25753_CMVideo00024	25753	9	F	218	3	0	0	0	0	0	Hospita	Test	Near	Visible-l	Unobstr	Small	Medical-1

											l			ight	ucted		080p
25786_CMVideo00423	25786	4.33	M	300	3	0	0	0	0	0	Hospital	Test	Near	Visible-light	Obstructed	Small	Medical-1080p
25698_CMVideo00371	25698	14	M	300	2	1	1	1	0	0	Hospital	Train	Near	Visible-light	Obstructed	Large	Medical-1080p
WUSI_20240412_105332_002	WUSI	35	F	300	3	1	1	1	1	1	Home	Train	Near	Visible-light	Obstructed	Large	Consumer-720p
WUSI_20240412_105332_001	WUSI	35	F	300	3	1	1	1	1	1	Home	Train	Near	Visible-light	Obstructed	Large	Consumer-720p
WUSI_20240412_053928_002	WUSI	35	F	295	3	1	1	1	1	1	Home	Train	Near	Night-vision	Obstructed	Large	Consumer-720p
WUSI_20240412_053928_001	WUSI	35	F	300	3	1	1	1	1	1	Home	Train	Near	Night-vision	Obstructed	Large	Consumer-720p
WUSI_20240412_015955_002	WUSI	35	F	272	3	0	1	1	1	1	Home	Train	Near	Night-vision	Obstructed	Large	Consumer-720p
WUSI_20240412_015955(1)_001	WUSI	35	F	153	3	0	0	1	0	1	Home	Train	Near	Night-vision	Obstructed	Small	Consumer-720p
WUSI_20240412_015955(2)_001	WUSI	35	F	94	3	0	1	1	1	1	Home	Train	Near	Visible-light	Obstructed	Large	Consumer-720p
HUXU_20230125_170135_001	HUXU	33	F	131	4	0	0	0	1	0	Home	Test	Near	Visible-light	Obstructed	Small	Consumer-720p
HUXU_20240125_093921_001	HUXU	33	F	92	4	0	0	0	1	0	Home	Test	Near	Visible-light	Obstructed	Small	Consumer-720p
YXDO_20231108_202929_203229	YXDO	25	M	179	3	1	1	1	0	1	Home	Train	Far	Night-vision	Obstructed	Large	Consumer-720p
WJQI_20231004_004125	WJQI	30	M	102	2	1	1	1	0	1	Home	Train	Near	Night-vi	Obstruct	Large	Consumer

_004307_02														sion	ed		-720p
WJQI_20231004_004125_004307_01	WJQI	30	M	102	2	1	1	1	0	0	Home	Train	Near	Night-vision	Obstructed	Large	Consumer-720p
WJQI_20230927_053755_053921_01	WJQI	30	M	86	2	0	1	0	0	0	Home	Train	Near	Night-vision	Obstructed	Large	Consumer-720p
WJQI_20230927_053755_053921_02	WJQI	30	M	86	2	0	1	0	0	0	Home	Train	Near	Night-vision	Obstructed	Large	Consumer-720p
WJQI_20231023_005808_010004_02	WJQI	30	M	116	2	1	1	1	0	1	Home	Train	Near	Night-vision	Obstructed	Large	Consumer-720p
WJQI_20231023_005808_010004_01	WJQI	30	M	116	2	1	1	1	0	0	Home	Train	Near	Night-vision	Obstructed	Large	Consumer-720p
JCYO_20231030_230821	JCYO	34	M	39	4	1	0	0	0	0	Home	Test	Far	Visible-light	Unobstructed	Large	Consumer-720p
JCYO_20231022_220955	JCYO	34	M	32	4	1	0	0	0	0	Home	Test	Far	Visible-light	Unobstructed	Large	Consumer-720p
JCYO_20230811_224308_224328_2	JCYO	34	M	21	4	1	0	0	0	0	Home	Test	Far	Visible-light	Unobstructed	Large	Consumer-720p
JCYO_20230814_140303_140319_2	JCYO	34	M	16	4	1	0	0	0	0	Home	Test	Far	Visible-light	Unobstructed	Large	Consumer-720p
WJQI_20231023_005808_010004_03	WJQI	30	M	116	2	0	1	0	0	0	Home	Train	Far	Night-vision	Obstructed	Large	Consumer-720p
WJQI_20231004_004125_004307_03	WJQI	30	M	102	2	1	1	0	0	0	Home	Train	Far	Night-vision	Obstructed	Large	Consumer-720p
YADA_20240330_061325	YADA	35	F	19	2	1	1	0	0	0	Home	Test	Far	Night-vision	Obstructed	Large	Consumer-720p
YADA_20240331_01245	YAD	35	F	57	2	1	1	0	0	0	Home	Test	Far	Night-vi	Obstruct	Large	Consumer

8	A													sion	ed		-720p
YADA_20240402_021816	YADA	35	F	60	2	1	1	0	0	0	Home	Test	Far	Night-vision	Obstructed	Large	Consumer-720p
YADA_20240403_023253	YADA	35	F	81	2	1	0	0	0	0	Home	Test	Far	Night-vision	Obstructed	Large	Consumer-720p
JCYO_20231019_065143	JCYO	34	M	34	4	1	1	0	0	0	Home	Test	Far	Night-vision	Obstructed	Large	Consumer-720p
JCYO_20231019_074941	JCYO	34	M	81	4	1	1	0	0	0	Home	Test	Far	Night-vision	Obstructed	Large	Consumer-720p
JCYO_20231111_050918	JCYO	34	M	64	4	1	0	0	0	0	Home	Test	Far	Night-vision	Obstructed	Small	Consumer-720p
JCYO_20230812_010402_010425_2	JCYO	34	M	24	4	1	0	0	0	0	Home	Test	Far	Night-vision	Unobstructed	Small	Consumer-720p
JCYO_20230812_141839_141857_2	JCYO	34	M	19	4	1	1	0	0	0	Home	Test	Far	Visible-light	Unobstructed	Small	Consumer-720p
JCYO_20230813_152411_152423_2	JCYO	34	M	13	4	1	0	0	0	0	Home	Test	Far	Night-vision	Unobstructed	Small	Consumer-720p
JCYO_20230813_164738_164753_2	JCYO	34	M	16	4	1	0	0	0	0	Home	Test	Far	Night-vision	Unobstructed	Large	Consumer-720p
JCYO_20230814_140303_140320_2	JCYO	34	M	17	4	1	0	0	0	0	Home	Test	Far	Night-vision	Unobstructed	Large	Consumer-720p
JCYO_20231103_022037	JCYO	34	M	38	4	1	0	0	0	0	Home	Test	Far	Night-vision	Unobstructed	Large	Consumer-720p
JCYO_20231124_020512	JCYO	34	M	29	4	1	0	0	0	0	Home	Test	Far	Night-vision	Unobstructed	Small	Consumer-720p
JCYO_20231119_012202	JCYO	34	M	51	4	0	1	0	0	0	Home	Test	Far	Night-vi	Unobstr	Large	Consumer

														sion	ucted		-720p
333_HX001	333	26	M	300	3	1	1	1	0	0	Hospital	External-Center2	NA	NA	NA	NA	NA
805_HX002	805	6.33	M	300	1	1	1	1	0	0	Hospital	External-Center2	NA	NA	NA	NA	NA
251_HX003	251	4.33	F	300	3	0	0	0	1	0	Hospital	External-Center2	NA	NA	NA	NA	NA
230_HX004	230	4.33	F	300	3	0	0	0	0	1	Hospital	External-Center2	NA	NA	NA	NA	NA
675_HX005	675	2.92	F	300	2	0	0	0	0	1	Hospital	External-Center2	NA	NA	NA	NA	NA
128_HX006	128	2.33	F	300	4	0	0	0	1	1	Hospital	External-Center2	NA	NA	NA	NA	NA
128_HX007	128	2.33	F	300	4	0	0	0	1	1	Hospital	External-Center2	NA	NA	NA	NA	NA
128_HX008	128	2.33	F	48	4	0	0	0	1	1	Hospital	External-Center2	NA	NA	NA	NA	NA
128_HX009	128	2.33	F	300	4	0	0	0	1	1	Hospital	External-Center2	NA	NA	NA	NA	NA
128_HX010	128	2.33	F	300	4	0	0	0	1	1	Hospital	External-Center2	NA	NA	NA	NA	NA
400_HX011	400	8	M	194	3	0	0	0	1	1	Hospital	External-Center2	NA	NA	NA	NA	NA
400_HX012	400	8	M	300	3	0	0	0	1	1	Hospital	External-Center2	NA	NA	NA	NA	NA
847_HX013	847	35	F	300	3	1	1	1	0	0	Hospita	External-C	NA	NA	NA	NA	NA

											l	enter2					
298_HX016	298	6	F	300	4	1	0	0	0	0	Hospital	External-Center2	NA	NA	NA	NA	NA
968_HX017	968	24	M	300	2	1	1	1	0	0	Hospital	External-Center2	NA	NA	NA	NA	NA
821_HX018	821	30	M	300	2	1	0	1	0	0	Hospital	External-Center2	NA	NA	NA	NA	NA
982_HX019	982	50	F	132	3	1	1	1	0	0	Hospital	External-Center2	NA	NA	NA	NA	NA
475_HX020	475	15	F	131	5	1	1	1	0	0	Hospital	External-Center2	NA	NA	NA	NA	NA
475_HX021	475	15	F	300	5	1	1	1	0	0	Hospital	External-Center2	NA	NA	NA	NA	NA
479_HX022	479	10	F	300	1	1	1	1	0	0	Hospital	External-Center2	NA	NA	NA	NA	NA
400_HX025	400	8	M	300	3	0	0	0	1	1	Hospital	External-Center2	NA	NA	NA	NA	NA
982_HX027	982	50	F	300	3	1	1	1	0	0	Hospital	External-Center2	NA	NA	NA	NA	NA
475_HX028	475	15	F	300	5	1	1	1	0	0	Hospital	External-Center2	NA	NA	NA	NA	NA
455_HX029	455	28	M	300	3	1	1	1	0	0	Hospital	External-Center2	NA	NA	NA	NA	NA
857_HX030	857	10.42	F	300	2	1	1	1	0	0	Hospital	External-Center2	NA	NA	NA	NA	NA
821_HX031	821	30	M	300	2	1	0	1	0	0	Hospita	External-C	NA	NA	NA	NA	NA

											l	enter2					
320_HX032	320	13	F	300	2	1	1	1	0	0	Hospital	External-Center2	NA	NA	NA	NA	NA
576_HX033	576	32	M	300	2	0	0	0	0	0	Hospital	External-Center2	NA	NA	NA	NA	NA
172_HX034	172	3	F	300	4	0	0	0	0	0	Hospital	External-Center2	NA	NA	NA	NA	NA
679_HX035	679	5.08	F	300	0	0	0	0	0	0	Hospital	External-Center2	NA	NA	NA	NA	NA
721_HX036	721	6.83	M	300	1	0	0	0	0	0	Hospital	External-Center2	NA	NA	NA	NA	NA
918_HX037	918	5	M	300	3	0	0	0	0	0	Hospital	External-Center2	NA	NA	NA	NA	NA
156_HX038	156	2.33	M	300	2	0	0	0	0	0	Hospital	External-Center2	NA	NA	NA	NA	NA
922_HX039	922	6	F	300	3	0	0	0	0	0	Hospital	External-Center2	NA	NA	NA	NA	NA
347_HX040	347	15	F	300	5	0	0	0	0	0	Hospital	External-Center2	NA	NA	NA	NA	NA
981_HX041	981	5	F	300	2	0	0	0	0	0	Hospital	External-Center2	NA	NA	NA	NA	NA
443_HX042	443	18	F	300	1	0	0	0	0	0	Hospital	External-Center2	NA	NA	NA	NA	NA
862_HX043	862	4.5	F	300	1	0	0	0	0	0	Hospital	External-Center2	NA	NA	NA	NA	NA
263_HX044	263	4	M	300	2	0	0	0	0	0	Hospita	External-C	NA	NA	NA	NA	NA

											l	enter2					
201_HX045	201	28	F	300	0	0	0	0	0	0	Hospital	External-Center2	NA	NA	NA	NA	NA
866_HX046	866	10.8	M	300	1	0	0	0	0	0	Hospital	External-Center2	NA	NA	NA	NA	NA
271_HX047	271	10.8	M	300	1	0	0	0	0	0	Hospital	External-Center2	NA	NA	NA	NA	NA
848_HX048	848	9.67	M	300	0	0	0	0	0	0	Hospital	External-Center2	NA	NA	NA	NA	NA
115_HX049	115	12.83	M	300	3	0	0	0	0	0	Hospital	External-Center2	NA	NA	NA	NA	NA
719_HX050	719	6.95	M	300	3	0	0	0	0	0	Hospital	External-Center2	NA	NA	NA	NA	NA
237_HX051	237	15	M	300	2	0	0	0	0	0	Hospital	External-Center2	NA	NA	NA	NA	NA
217_HX052	217	4	M	300	2	0	0	0	0	0	Hospital	External-Center2	NA	NA	NA	NA	NA
356_HX053	356	12.5	M	300	1	0	0	0	0	0	Hospital	External-Center2	NA	NA	NA	NA	NA
564_HX054	564	10.83	F	300	2	0	0	0	0	0	Hospital	External-Center2	NA	NA	NA	NA	NA
537_HX055	537	8.75	M	300	1	0	0	0	0	0	Hospital	External-Center2	NA	NA	NA	NA	NA
142_HX056	142	2	F	300	3	0	0	0	0	0	Hospital	External-Center2	NA	NA	NA	NA	NA
830_HX057	830	24	M	300	1	0	0	0	0	0	Hospita	External-C	NA	NA	NA	NA	NA

											l	enter2					
899_HX058	899	33	M	300	1	0	0	0	0	0	Hospital	External-Center2	NA	NA	NA	NA	NA
892_HX059	892	23	M	300	1	0	0	0	0	0	Hospital	External-Center2	NA	NA	NA	NA	NA
972_HX060	972	5	M	300	3	0	0	0	0	0	Hospital	External-Center2	NA	NA	NA	NA	NA
617_HX061	617	46	F	300	4	0	0	0	0	0	Hospital	External-Center2	NA	NA	NA	NA	NA
pat01_Sz1PG	pat01	28	M	300	-	1	0	1	0	0	Hospital	External-Public	NA	NA	NA	NA	NA
pat01_Sz2PG	pat01	28	M	836	-	1	1	0	0	0	Hospital	External-Public	NA	NA	NA	NA	NA
pat02_Sz1PG	pat02	37	M	306	-	1	1	1	0	0	Hospital	External-Public	NA	NA	NA	NA	NA
pat02_Sz2PG	pat02	37	M	300	-	1	1	1	0	0	Hospital	External-Public	NA	NA	NA	NA	NA
pat03_Sz2PG	pat03	39	M	82	-	1	1	1	0	0	Hospital	External-Public	NA	NA	NA	NA	NA
pat07_Sz1PG	pat07	32	M	258	-	1	1	0	0	0	Hospital	External-Public	NA	NA	NA	NA	NA
pat07_Sz2PG	pat07	32	M	240	-	1	1	0	0	0	Hospital	External-Public	NA	NA	NA	NA	NA
pat09_Sz1P	pat09	31	M	263	-	0	0	0	1	0	Hospital	External-Public	NA	NA	NA	NA	NA
pat09_Sz2P	pat09	31	M	361	-	1	0	0	1	0	Hospita	External-P	NA	NA	NA	NA	NA

											l	ublic					
pat09_Sz3P	pat09	31	M	236	-	1	0	0	0	0	Hospital	External-Public	NA	NA	NA	NA	NA
pat10_Sz1P	pat10	24	F	35	-	1	0	0	0	0	Hospital	External-Public	NA	NA	NA	NA	NA
pat10_Sz2P	pat10	24	F	32	-	1	0	0	0	0	Hospital	External-Public	NA	NA	NA	NA	NA
pat11_Sz1P	pat11	55	F	152	-	0	0	0	1	0	Hospital	External-Public	NA	NA	NA	NA	NA
pat12_Sz1P	pat12	23	M	225	-	0	0	0	1	0	Hospital	External-Public	NA	NA	NA	NA	NA
pat12_Sz2P	pat12	23	M	27	-	0	0	0	1	0	Hospital	External-Public	NA	NA	NA	NA	NA
pat12_Sz3P	pat12	23	M	52	-	0	0	0	1	0	Hospital	External-Public	NA	NA	NA	NA	NA
pat12_Sz4P	pat12	23	M	93	-	0	0	0	1	0	Hospital	External-Public	NA	NA	NA	NA	NA
pat13_Sz1PG	pat13	29	F	92	-	1	1	0	1	0	Hospital	External-Public	NA	NA	NA	NA	NA
pat14_Sz1PG	pat14	18	M	146	-	1	1	0	0	0	Hospital	External-Public	NA	NA	NA	NA	NA
pat14_Sz2PG	pat14	18	M	150	-	1	1	0	0	0	Hospital	External-Public	NA	NA	NA	NA	NA

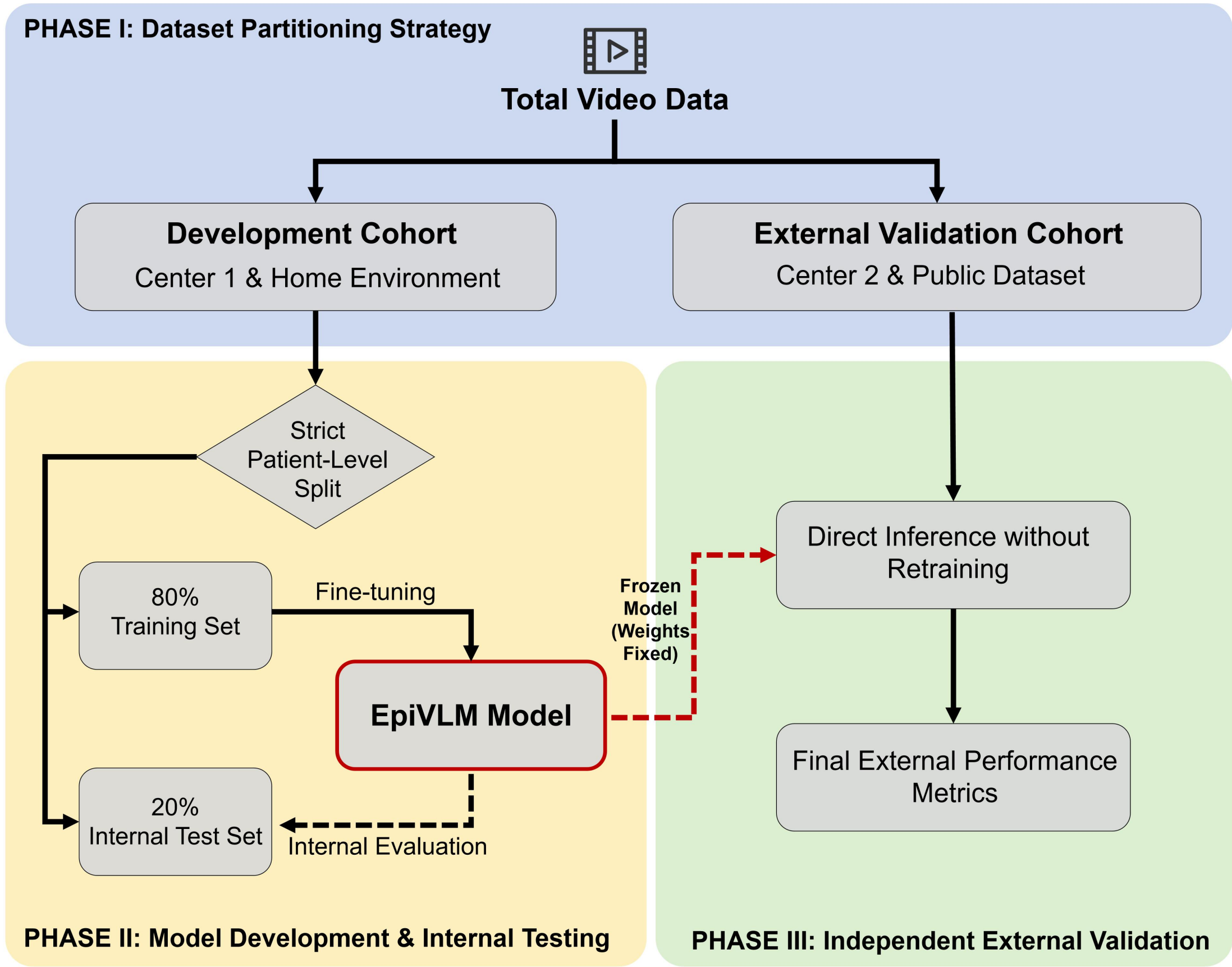

Figure S1. Data workflow for model fine-tuning, testing, and validation. Phase I: Dataset Partitioning: Total video data were divided into a Development Cohort (Center 1 & Home) and an External Validation Cohort (Center 2 & public dataset). Phase II: Model Development & Internal Testing: Within the development cohort, a strict patient-level split was applied; 80% of videos were used for fine-tuning and 20% for internal evaluation. Phase III: Independent External Validation: The fine-tuned model was applied to the external cohort without retraining to compute final performance metrics.

Table S2. Direct-question prompt templates for model inference

Semiology	Stage / Step	Role	Prompt text
Clonic	System prompt	System	You are an expert in epilepsy.
Clonic	Direct question	User	Is the patient experiencing a clonic seizure in this video? Answer Yes or No.
Tonic	System prompt	System	You are an expert in epilepsy.
Tonic	Direct question	User	Is the patient experiencing a tonic seizure in this video? Answer Yes or No.
Versive	System prompt	System	You are an expert in epilepsy.
Versive	Direct question	User	Is the patient experiencing a versive seizure in this video? Answer Yes or No.
Manual automatisms	System prompt	System	You are an expert in epilepsy.
Manual automatisms	Direct question	User	Is the patient showing manual automatisms in this video? Answer Yes or No.
Staring	System prompt	System	You are an expert in epilepsy.
Staring	Direct question	User	Is the patient having a staring episode in this video? Answer Yes or No.

Table S3. Semiology-specific prompt templates for EpiVLM inference

Semiology	Stage / Step	Role	Prompt text
Clonic	System prompt	System	You are an expert in epilepsy.
Clonic	Step 1 – Observation prompt	User	Observe the epileptic's hand movements and don't miss any tiny details. Answer concisely like this: 1. Right Hand: ... 2. Left Hand: ... 3. Overall Movement: ...
Clonic	Step 2 – Decision prompt	User	Strictly follow the instructions: During a mild clonic seizure, … Tell me: clonic seizure = True/False. Answer concisely.
Tonic	System prompt	System	You are an expert in epilepsy.
Tonic	Step 1 – Observation prompt	User	Observe the patient in the video. Are there any abnormal postures…? Please answer concisely in one sentence.
Versive	System prompt	System	You are an expert in epilepsy.
Versive	Step 1 – Observation prompt	User	Observe the patient lying in the bed in the consecutive images. Does the epilepsy patient show subtle deviation (versive)…? Please answer concisely in one sentence.
Manual automatisms	System prompt	System	You are an expert in epilepsy.
Manual automatisms	Step 1 – Observation prompt	User	Observe the patient in the video, are there any subtle hand movements? Answer concisely.
Manual automatisms	Step 2 – Decision prompt	User	Strictly follow the instructions: During seizures, patients may exhibit only subtle symptoms… Tell me: hand automatism = True/False.
Staring	System prompt	System	You are an expert in epilepsy.
Staring	Step 1 – Observation prompt	User	Observe the patient in the video. Are the patient’s eyes open…? Please answer concisely in one sentence.
Staring	Step 2 – Decision prompt	User	Previous answer: {first_answer} Instruction: If the previous answer contains 'Yes', reply exactly 'staring = True'. If the previous answer contains 'No', reply exactly 'staring = False'. Do not provide any explanation or additional interpretation.

Table S4. Performance metrics for seizure semiology recognition by symptom and evaluation type

Type	Symptom	Accuracy (95%CI)	Precision (95%CI)	Sensitivity (95%CI)	F1 (95%CI)	Specificity (95%CI)	FPR (95%CI)
Direct-question prompting	Clonic	0.757(0.721, 0.791)	0.894(0.806, 0.968)	0.279(0.216, 0.343)	0.424(0.345, 0.502)	0.984(0.970, 0.995)	0.016(0.005, 0.030)
	Tonic	0.779(0.747, 0.813)	0.893(0.760, 1.000)	0.161(0.106, 0.222)	0.271(0.190, 0.357)	0.993(0.984, 1.000)	0.007(0.000, 0.016)
	Versive	0.750(0.717, 0.784)	0.635(0.300, 0.917)	0.043(0.013, 0.077)	0.080(0.025, 0.139)	0.992(0.983, 0.998)	0.008(0.002, 0.017)
	Manual automatisms	0.497(0.462, 0.530)	0.768(0.705, 0.834)	0.276(0.237, 0.319)	0.406(0.358, 0.458)	0.862(0.820, 0.902)	0.138(0.098, 0.180)
	Staring	0.352(0.326, 0.380)	0.789(0.674, 0.902)	0.046(0.032, 0.062)	0.087(0.062, 0.116)	0.975(0.960, 0.989)	0.025(0.011, 0.040)
Clinically structured prompting	Clonic	0.863(0.835, 0.890)	0.767(0.707, 0.827)	0.827(0.767, 0.879)	0.795(0.751, 0.839)	0.880(0.846, 0.911)	0.120(0.089, 0.154)
	Tonic	0.886(0.861, 0.913)	0.839(0.776, 0.899)	0.696(0.623, 0.766)	0.760(0.704, 0.816)	0.953(0.934, 0.971)	0.047(0.029, 0.066)
	Versive	0.889(0.864, 0.911)	0.788(0.721, 0.847)	0.774(0.708, 0.836)	0.780(0.732, 0.826)	0.929(0.906, 0.949)	0.071(0.051, 0.094)
	Manual automatisms	0.768(0.734, 0.800)	0.794(0.757, 0.828)	0.850(0.817, 0.883)	0.821(0.793, 0.847)	0.634(0.573, 0.690)	0.366(0.310, 0.427)
	Staring	0.897(0.878, 0.915)	0.911(0.891, 0.930)	0.939(0.923, 0.954)	0.924(0.909, 0.938)	0.813(0.775, 0.851)	0.187(0.149, 0.225)
Supervised fine-tuning	Clonic	0.889(0.863, 0.916)	0.776(0.719, 0.830)	0.927(0.888, 0.962)	0.844(0.804, 0.881)	0.870(0.836, 0.903)	0.130(0.097, 0.164)
	Tonic	0.904(0.881, 0.927)	0.794(0.737, 0.854)	0.854(0.798, 0.906)	0.823(0.779, 0.864)	0.922(0.897, 0.946)	0.078(0.054, 0.103)
	Versive	0.897(0.872, 0.919)	0.792(0.736, 0.848)	0.848(0.794, 0.900)	0.819(0.773, 0.858)	0.915(0.890, 0.939)	0.085(0.061, 0.110)
	Manual automatisms	0.795(0.764, 0.823)	0.802(0.764, 0.836)	0.893(0.864, 0.920)	0.845(0.819, 0.867)	0.634(0.574, 0.691)	0.366(0.309, 0.426)
	Staring	0.942(0.930, 0.955)	0.966(0.954, 0.979)	0.947(0.932, 0.962)	0.957(0.946, 0.966)	0.932(0.907, 0.957)	0.068(0.043, 0.093)

Table S5. Performance improvement in adverse visual conditions using temporal post-processing

Data Subset	Metric	Original	Post-processed	Improvement
720p Videos	Specificity	68.81%	71.56%	**+ 2.75%**
	Recall	89.36%	89.36%	**Maintained**
Night-Vision	Specificity	73.68%	77.63%	**+ 3.95%**
	Recall	86.87%	86.87%	**Maintained**

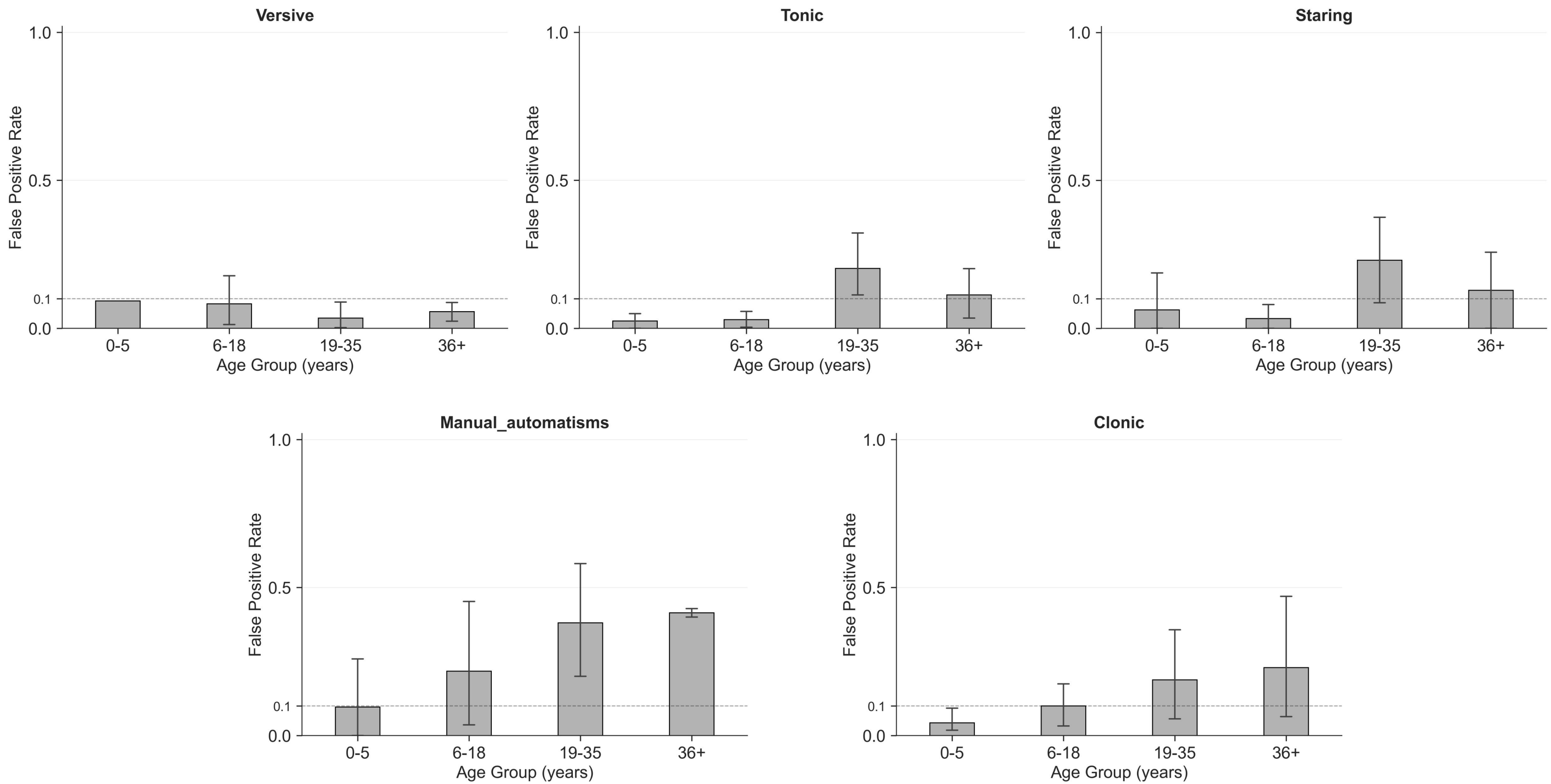

Figure S2. False Positive Rate (FPR) stratified by age group and symptoms. Bar plots show the FPR of the EpiVLM model for each seizure type (versive, tonic, staring, manual automatisms, clonic) stratified by four age groups (0 – 5, 6 – 18, 19 – 35, ⩾36 years).

Table S6. Age group performance details

Age Group	Symptom	Accuracy (95%CI)	Precision (95%CI)	Sensitivity (95%CI)	F1 (95%CI)	Specificity (95%CI)	FPR (95%CI)
0-5	Clonic	0.949 (0.915, 0.966)	0.667 (0.500, 0.750)	0.833 (0.750, 1.000)	0.722 (0.667, 0.750)	0.957 (0.907, 0.982)	0.043 (0.018, 0.093)
0-5	Manual_automatisms	0.875 (0.771, 0.952)	0.946 (0.865, 1.000)	0.902 (0.799, 0.970)	0.909 (0.830, 0.972)	0.267 (0.060, 0.534)	0.096 (0.000, 0.259)
0-5	Staring	0.983 (0.969, 0.996)	0.935 (0.808, 1.000)	0.923 (0.792, 0.996)	0.929 (0.801, 0.996)	0.125 (0.000, 0.312)	0.062 (0.000, 0.188)
0-5	Tonic	0.982 (0.964, 1.000)	0.941 (0.882, 1.000)	1.000 (1.000, 1.000)	0.969 (0.938, 1.000)	0.975 (0.950, 1.000)	0.025 (0.000, 0.050)
0-5	Versive	0.917 (N/A)	0.545 (N/A)	1.000 (N/A)	0.706 (N/A)	0.907 (N/A)	0.093 (N/A)
6-18	Clonic	0.910 (0.874, 0.947)	0.808 (0.729, 0.890)	0.932 (0.841, 0.989)	0.854 (0.831, 0.876)	0.900 (0.825, 0.968)	0.100 (0.032, 0.175)
6-18	Manual_automatisms	0.878 (0.788, 0.957)	0.795 (0.542, 0.975)	0.790 (0.534, 0.959)	0.789 (0.532, 0.961)	0.283 (0.067, 0.565)	0.217 (0.036, 0.453)
6-18	Staring	0.932 (0.880, 0.977)	0.735 (0.486, 0.943)	0.732 (0.482, 0.931)	0.725 (0.478, 0.934)	0.367 (0.085, 0.650)	0.033 (0.000, 0.081)
6-18	Tonic	0.943 (0.920, 0.967)	0.797 (0.622, 0.971)	0.837 (0.677, 0.978)	0.781 (0.700, 0.879)	0.971 (0.943, 0.996)	0.029 (0.004, 0.057)
6-18	Versive	0.897 (0.808, 0.958)	0.794 (0.646, 0.937)	0.813 (0.720, 0.912)	0.787 (0.685, 0.889)	0.918 (0.823, 0.987)	0.082 (0.013, 0.177)
19-35	Clonic	0.861 (0.784, 0.921)	0.861 (0.776, 0.933)	0.887 (0.806, 0.953)	0.854 (0.801, 0.901)	0.669 (0.449, 0.865)	0.188 (0.056, 0.357)
19-35	Manual_automatisms	0.761 (0.656, 0.844)	0.746 (0.610, 0.858)	0.861 (0.774, 0.935)	0.783 (0.665, 0.860)	0.519 (0.303, 0.727)	0.381 (0.200, 0.581)
19-35	Staring	0.800 (0.700, 0.917)	0.562 (0.286, 0.925)	0.848 (0.712, 1.000)	0.618 (0.444, 0.804)	0.770 (0.625, 0.914)	0.230 (0.086, 0.375)
19-35	Tonic	0.879 (0.834, 0.918)	0.857 (0.805, 0.906)	0.899 (0.846, 0.948)	0.861 (0.824, 0.898)	0.642 (0.492, 0.774)	0.202 (0.113, 0.322)
19-35	Versive	0.948 (0.906, 0.977)	0.909 (0.804, 0.988)	0.870 (0.809, 0.927)	0.874 (0.810, 0.925)	0.965 (0.911, 0.998)	0.035 (0.002, 0.089)
36+	Clonic	0.863 (0.793, 0.923)	0.855 (0.787, 0.928)	0.851 (0.673, 0.976)	0.826 (0.714, 0.910)	0.771 (0.530, 0.936)	0.229 (0.064, 0.470)
36+	Manual_automatisms	0.682 (0.525, 0.839)	0.448 (0.062, 0.833)	0.576 (0.200, 0.952)	0.492 (0.095, 0.889)	0.586 (0.571, 0.600)	0.414 (0.400, 0.429)
36+	Staring	0.917 (0.833, 1.000)	0.364 (0.000, 0.727)	0.480 (0.000, 0.960)	0.414 (0.000, 0.828)	0.871 (0.743, 1.000)	0.129 (0.000, 0.257)
36+	Tonic	0.855 (0.801, 0.914)	0.849 (0.725, 0.951)	0.823 (0.723, 0.918)	0.813 (0.737, 0.885)	0.788 (0.594, 0.941)	0.112 (0.035, 0.201)
36+	Versive	0.926 (0.907, 0.949)	0.760 (0.623, 0.887)	0.838 (0.753, 0.906)	0.795 (0.678, 0.895)	0.944 (0.913, 0.976)	0.056 (0.024, 0.087)

Table S7. Performance of two human raters and EpiVLM by seizure semiology

Symptom	κ (95% CI)	F1			FPR			Accuracy			Sensitivity			Specificity		
		Doc1	Doc2	EpiVLM	Doc1	Doc2	EpiVLM	Doc1	Doc2	EpiVLM	Doc1	Doc2	EpiVLM	Doc1	Doc2	EpiVLM
Clonic	0.981 (0.976-0.987)	0.987 (0.982-0.991)	0.985 (0.980-0.989)	0.856 (0.824-0.885)	0.004 (0.003-0.005)	0.004 (0.003-0.005)	0.093 (0.059-0.133)	0.996 (0.995-0.998)	0.996 (0.995-0.997)	0.906 (0.882-0.927)	0.997 (0.994-0.999)	0.994 (0.990-0.997)	0.904 (0.856-0.941)	0.996 (0.995-0.997)	0.996 (0.995-0.997)	0.907 (0.867-0.941)
Manual Automatisms	0.951 (0.942-0.960)	0.972 (0.965-0.978)	0.971 (0.964-0.977)	0.884 (0.822-0.930)	0.003 (0.002-0.005)	0.002 (0.001-0.003)	0.291 (0.172-0.436)	0.993 (0.992-0.995)	0.993 (0.991-0.995)	0.834 (0.768-0.892)	0.967 (0.957-0.976)	0.959 (0.947-0.969)	0.883 (0.819-0.937)	0.997 (0.995-0.998)	0.998 (0.997-0.999)	0.709 (0.564-0.828)
Staring	0.988 (0.984-0.991)	0.992 (0.989-0.995)	0.990 (0.986-0.993)	0.960 (0.934-0.980)	0.002 (0.001-0.003)	0.003 (0.002-0.004)	0.091 (0.034-0.166)	0.997 (0.996-0.998)	0.996 (0.995-0.997)	0.941 (0.911-0.968)	0.994 (0.991-0.997)	0.992 (0.988-0.996)	0.953 (0.920-0.980)	0.998 (0.997-0.999)	0.997 (0.996-0.998)	0.909 (0.834-0.966)
Tonic	0.975 (0.969-0.981)	0.983 (0.979-0.988)	0.982 (0.977-0.987)	0.850 (0.815-0.881)	0.004 (0.003-0.006)	0.003 (0.002-0.005)	0.065 (0.042-0.094)	0.995 (0.994-0.996)	0.995 (0.994-0.996)	0.918 (0.895-0.937)	0.993 (0.989-0.997)	0.985 (0.979-0.991)	0.871 (0.823-0.916)	0.996 (0.994-0.997)	0.997 (0.995-0.998)	0.935 (0.906-0.958)
Versive	0.950 (0.938-0.959)	0.964 (0.955-0.972)	0.965 (0.956-0.973)	0.849 (0.780-0.911)	0.004 (0.003-0.006)	0.003 (0.002-0.005)	0.054 (0.025-0.089)	0.993 (0.992-0.995)	0.994 (0.992-0.995)	0.929 (0.897-0.956)	0.970 (0.958-0.980)	0.964 (0.953-0.976)	0.871 (0.815-0.919)	0.996 (0.994-0.997)	0.997 (0.995-0.998)	0.946 (0.911-0.975)

Values are point estimates with 95% confidence intervals (CI). FPR = false positive rate.

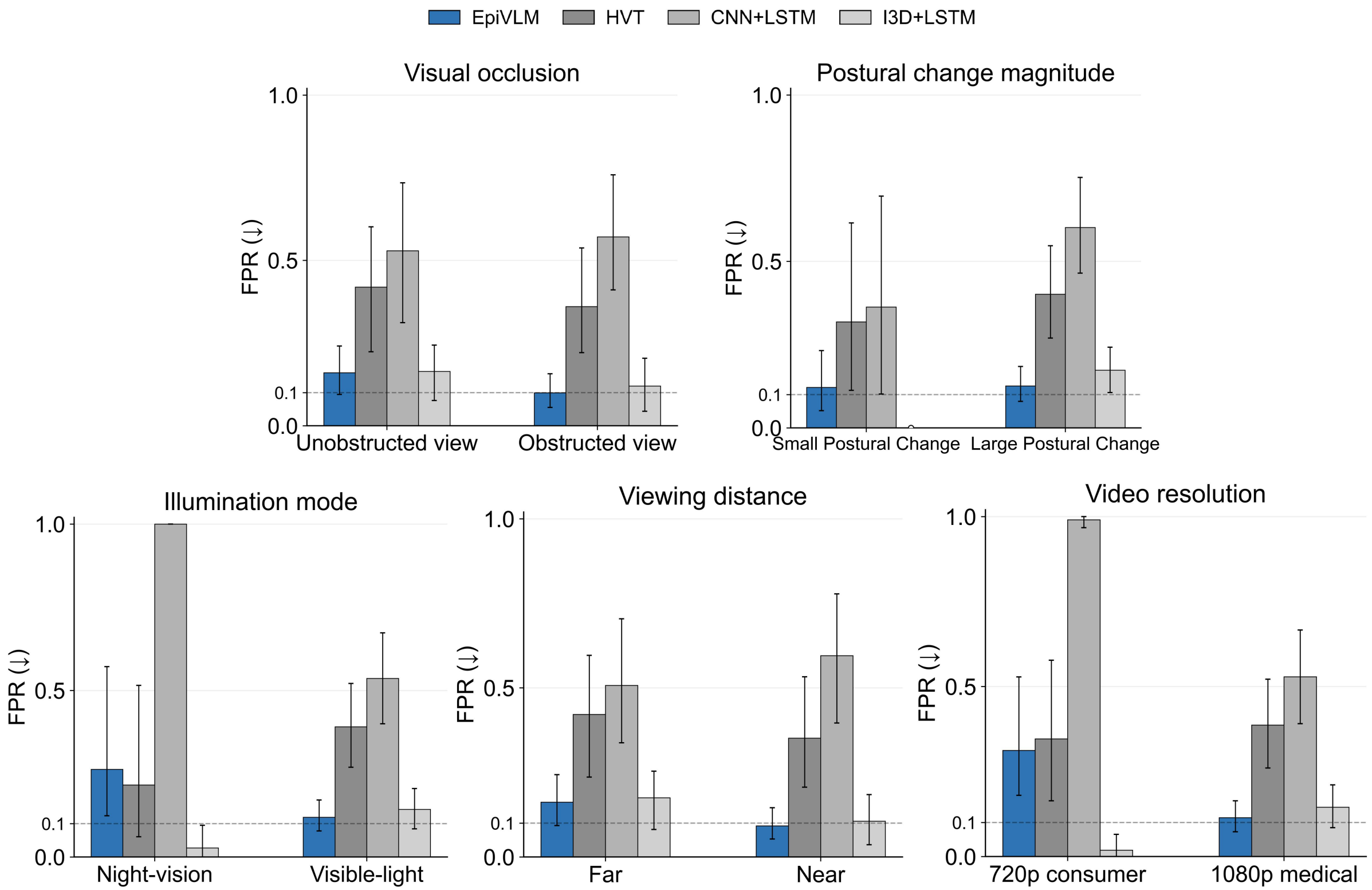

Figure S3. False Positive Rate (FPR) for four models under different recording conditions. Bar plots show FPR for four models (EpiVLM, HVT, CNN+LSTM, I3D+LSTM) across five recording conditions: visual occlusion (Unobstructed vs Obstructed), postural change magnitude (Small vs Large), illumination mode (Night-vision vs Visible-light), viewing distance (Far vs Near), and video resolution (720p consumer vs 1080p medical).

Table S8.Performance by recognition difficulty bin across models

Model	DiffBin	Detected rate	Accuracy	Precision	Recall	F1	Specificity	FPR
EpiVLM	High	1.000 (1.000, 1.000)	0.818 (0.761, 0.859)	0.840 (0.733, 0.924)	0.883 (0.797, 0.965)	0.861 (0.794, 0.902)	0.703 (0.478, 0.838)	0.297 (0.162, 0.522)
EpiVLM	Low	1.000 (1.000, 1.000)	0.876 (0.835, 0.916)	0.796 (0.712, 0.877)	0.879 (0.832, 0.926)	0.835 (0.777, 0.893)	0.875 (0.811, 0.924)	0.125 (0.076, 0.189)
EpiVLM	Medium	1.000 (1.000, 1.000)	0.923 (0.883, 0.959)	0.934 (0.855, 0.984)	0.937 (0.903, 0.964)	0.935 (0.884, 0.967)	0.902 (0.821, 0.968)	0.098 (0.032, 0.179)
HVT	High	1.000 (1.000, 1.000)	0.764 (0.673, 0.837)	0.811 (0.652, 0.934)	0.811 (0.706, 0.897)	0.811 (0.713, 0.873)	0.687 (0.447, 0.877)	0.313 (0.123, 0.553)
HVT	Low	1.000 (1.000, 1.000)	0.684 (0.584, 0.783)	0.533 (0.442, 0.647)	0.812 (0.660, 0.933)	0.644 (0.559, 0.729)	0.615 (0.444, 0.765)	0.385 (0.235, 0.556)
HVT	Medium	0.952 (0.857, 1.000)	0.705 (0.564, 0.831)	0.723 (0.500, 0.907)	0.780 (0.589, 0.924)	0.750 (0.567, 0.875)	0.606 (0.374, 0.831)	0.394 (0.169, 0.626)
CNN+LSTM	High	0.941 (0.824, 1.000)	0.620 (0.457, 0.797)	0.623 (0.459, 0.801)	0.988 (0.944, 1.000)	0.764 (0.626, 0.887)	0.010 (0.000, 0.035)	0.990 (0.965, 1.000)
CNN+LSTM	Low	0.684 (0.474, 0.895)	0.446 (0.306, 0.564)	0.333 (0.225, 0.443)	0.578 (0.340, 0.825)	0.423 (0.284, 0.539)	0.374 (0.209, 0.535)	0.626 (0.465, 0.791)
CNN+LSTM	Medium	0.905 (0.762, 1.000)	0.689 (0.543, 0.822)	0.728 (0.507, 0.918)	0.721 (0.499, 0.904)	0.725 (0.533, 0.860)	0.645 (0.404, 0.858)	0.355 (0.142, 0.596)
I3D+LSTM	High	0.059 (0.000, 0.176)	0.399 (0.213, 0.564)	0.800 (0.667, 1.000)	0.049 (0.000, 0.139)	0.092 (0.000, 0.234)	0.980 (0.929, 1.000)	0.020 (0.000, 0.071)
I3D+LSTM	Low	0.737 (0.526, 0.895)	0.744 (0.612, 0.846)	0.656 (0.539, 0.753)	0.568 (0.335, 0.809)	0.609 (0.427, 0.750)	0.839 (0.761, 0.907)	0.161 (0.093, 0.239)
I3D+LSTM	Medium	0.524 (0.333, 0.714)	0.692 (0.523, 0.845)	0.863 (0.655, 1.000)	0.546 (0.282, 0.796)	0.669 (0.407, 0.847)	0.886 (0.771, 1.000)	0.114 (0.000, 0.229)

Values are point estimates with 95% confidence intervals (CI). FPR = false positive rate.
